# Time-resolved single-cell analysis of *Brca1* associated mammary tumourigenesis reveals aberrant differentiation of luminal progenitors

**DOI:** 10.1038/s41467-021-21783-3

**Published:** 2021-03-09

**Authors:** Karsten Bach, Sara Pensa, Marija Zarocsinceva, Katarzyna Kania, Julie Stockis, Silvain Pinaud, Kyren A. Lazarus, Mona Shehata, Bruno M. Simões, Alice R. Greenhalgh, Sacha J. Howell, Robert B. Clarke, Carlos Caldas, Timotheus Y. F. Halim, John C. Marioni, Walid T. Khaled

**Affiliations:** 1 University of Cambridge, Department of Pharmacology, Cambridge, UK; 2 Cancer Research UK Cambridge Institute, Li Ka Shing Centre, University of Cambridge, Cambridge, UK; 3 Cancer Research UK, Cambridge Cancer Centre, Cambridge, UK; 4 Wellcome-MRC Cambridge Stem Cell Institute, Cambridge, UK; 5 Medical Research Council Cancer Unit, University of Cambridge, Cambridge, UK; 6 Manchester Breast Centre, Oglesby Cancer Research Building, University of Manchester, Manchester, UK; 7 Department of Medical Oncology, Christie NHS Foundation Trust, Manchester, UK; 8 Wellcome Sanger Institute, Wellcome Genome Campus, Hinxton, Cambridge, UK; 9 European Bioinformatics Institute, European Molecular Biology Laboratory, Hinxton, UK

**Keywords:** Breast cancer, Cancer genomics

## Abstract

It is unclear how genetic aberrations impact the state of nascent tumour cells and their microenvironment. *BRCA1* driven triple negative breast cancer (TNBC) has been shown to arise from luminal progenitors yet little is known about how *BRCA1* loss-of-function (LOF) and concomitant mutations affect the luminal progenitor cell state. Here we demonstrate how time-resolved single-cell profiling of genetically engineered mouse models before tumour formation can address this challenge. We found that perturbing *Brca1/p53* in luminal progenitors induces aberrant alveolar differentiation pre-malignancy accompanied by pro-tumourigenic changes in the immune compartment. Unlike alveolar differentiation during gestation, this process is cell autonomous and characterised by the dysregulation of transcription factors driving alveologenesis. Based on our data we propose a model where *Brca1/p53* LOF inadvertently promotes a differentiation program hardwired in luminal progenitors, highlighting the deterministic role of the cell-of-origin and offering a potential explanation for the tissue specificity of *BRCA1* tumours.

## Abstract (web summary)

*BRCA1* driven breast cancer arises from luminal progenitor cells but how *BRCA1* loss-of-function affects the luminal progenitor cell state during premalignant stages of the disease is still unclear. Here, the authors demonstrate an aberrant differentiation of luminal progenitors towards a partial secretory luminal cell phenotype that occurs in a *Brca1* deficient mouse model of breast cancer at early stages of tumour initiation and in breast cells from *BRCA1* carriers.

## Introduction

One of the major hurdles for the early detection of cancer is our poor understanding of tumour-initiating events. Historically, cancer research has focused on histological and molecular characterisation of established tumours, which has led to the identification of hundreds of putative driver mutations. It is currently unclear how these genetic aberrations in tumour-initiating cells impact the cell state of nascent tumour cells and their microenvironment. *BRCA1*-driven triple-negative breast cancer (TNBC), for example, has been shown to arise from luminal progenitor cells^1,2^ yet little is known about how *BRCA1* loss-of-function (LOF) and concomitant mutations affect the luminal progenitor cell state and ultimately lead to transformation. To explore this in more detail, we used the *Brca1/p53* TNBC mouse model (*Blg-Cre; Brca1*^*f/f*^*;p53*^*+/*^^−^*)* that harbours a conditional *Brca1* LOF in the luminal progenitor compartment.

## Results

**Fig. 1:**
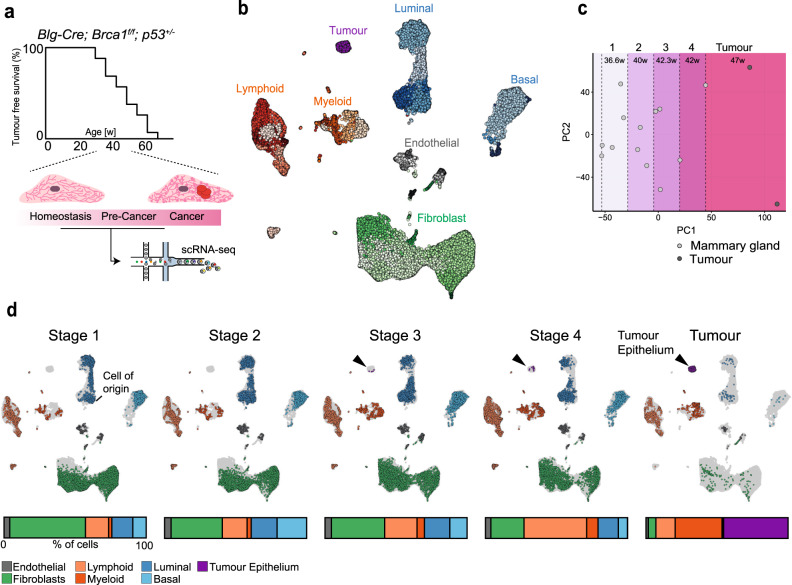
A time-resolved view of TNBC development in the *Blg-Cre; Brca1*^*f/f*^; p53^*+/−*^ mouse model at single-cell level. **a** Schematic overview of the experimental design. Mammary glands from 13 animals between 30 and 48 weeks of age as well as two fully developed tumours were prepared for scRNA sequencing after depleting dead cells. **b** UMAP of all samples, including wild-type controls, cells are coloured by cell type annotation. For the complete annotation see Supplementary Fig. 3b. **c** Principal component analysis computed on the pseudo-bulked, normalised and log-transformed counts from all samples of the *Blg-Cre; Brca1*^*f/f*^*; p53*^*+/*^^−^ animals. Dashed lines highlight the boundaries of the four stages pre-malignancy and the tumour stage. The mean age in each stage is noted at the top of the plot (Stage 1: 36.6w [30–41], Stage 2: 40w [38–41], Stage 3 42.3w [33–48], Stage 4: 42w [38–46], Tumour: 47w [46–48]). **d** UMAP from **b** subsetted by the stages identified in **c**. Cells are coloured by cell compartments. Grey cells in the background represent cells from all samples not present at the stage of interest. Bars underneath the UMAPs represent the tissue composition at each stage. PC principal component.

We performed single cell RNA sequencing (scRNA-seq) on cells isolated from the mammary glands of 15 *Brca1/p53* mice spanning various premalignant stages (*n* = 15) and fully developed tumours (*n* = 2) (Fig. 1a and Supplementary Fig. 1a, b). The dataset comprises ~100,000 cells that we grouped into 51 cell types/states spanning the epithelial, immune and stromal compartment (Fig. 1b and Supplementary Fig. 1c). Due to the lack of an external indicator of the samples’ premalignant stage we inferred the stages from the transcriptional data itself. For this, we pseudo-bulked the samples to derive a single transcriptional profile per sample and performed principal component analysis (PCA) to identify latent factors that drive variation in the data (Fig. 1c). We noted that PC1 appears to capture disease progression from wild-type like (low PC1 values) to fully developed tumours (high PC1 values). This was supported by a correlation of PC1 with age and was also reflected in the fact that genes with high loadings for PC1 were enriched for central processes of tumourigenesis (Supplementary Fig. 2). To facilitate the analysis, we divided the samples into four groups along PC1 (Stages 1–4) as well as one group of tumour samples (Fig. 1c). Despite the absence of visible tumours, we readily identified a small number of tumour cells in stages 3 and 4, highlighting the strength of the unbiased experimental and analytical approach (Fig. 1d).

**Fig. 2:**
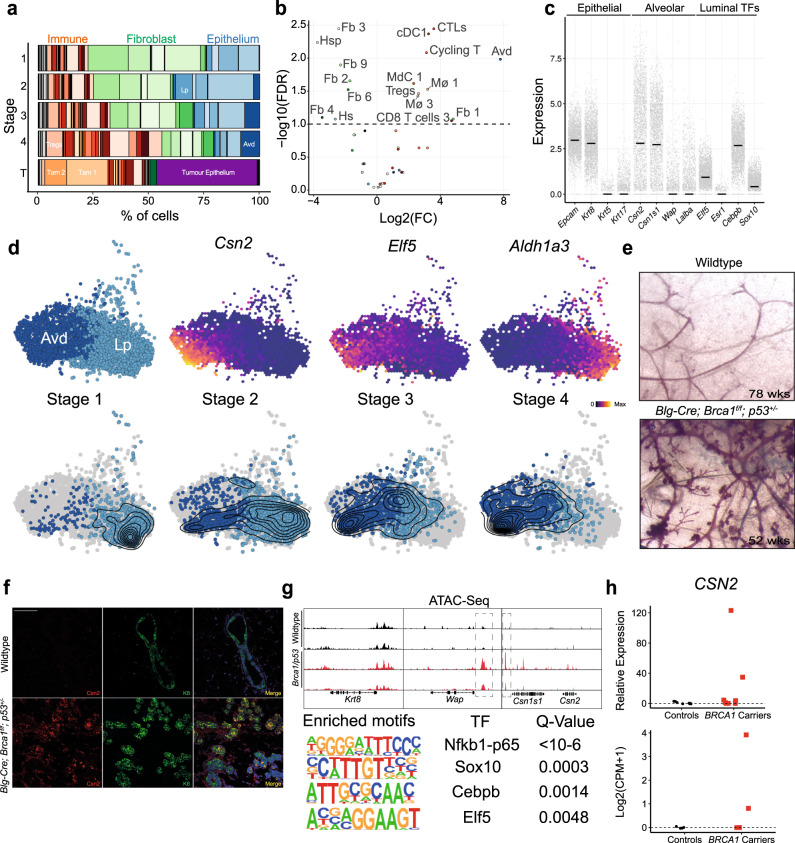
Luminal progenitor cells aberrantly differentiate towards an alveolar fate during *BRCA1* LOF-dependent TNBC development. **a** Cell type composition of all *Blg-Cre; Brca1*^*f/f*^*; p53*^*+/−*^ samples grouped by stages. Key cell types are highlighted, for full annotation see Supplementary Fig. 3a. **b** Volcano plot showing the results of the differential abundance test during tumour development from stage 1 to 4. The logFC represents the coefficient of a robust regression of normalised log-transformed cell type abundance on the 0–1 scaled PC1 values from Fig. 1c. Colour scheme corresponds to **a** and Supplementary Fig. 3. **c** Gene expression of various lineage-markers for the Avd cluster. Expression values represent normalised, log-transformed counts. The horizontal line depicts the median expression. Expression values are derived from *n* = 15 independent animals. **d** UMAP coordinates from Fig. 1, only showing the Lp and Avd cluster. The top row highlights the location of the two clusters as well as gene expression of three marker genes. The bottom row is facetted by stages with overlaid density estimate. **e** Wholemounts of mammary glands from wild-type and *Blg-Cre; Brca1*^*f/f*^*; p53*^*+/−*^ animals. Weeks (wks) of age are shown in the bottom right corner. Additional examples are shown in Supplementary Fig. 3c. **f** Immunofluorescence staining for Csn2 (red), Cytokeratin-8 (K8, green) and DAPI (blue) from wild-type (top row) and *Blg-Cre; Brca1*^*f/f*^*; p53*^*+/−*^ (bottom row) mammary glands. Scale bars represent 100 µm. Ten individual images from three independent animals were analysed. **g** ATAC-sequencing data from sorted luminal progenitor cells of wild-type (top) and *Blg-Cre; Brca1*^*f/f*^*; p53*^*+/−*^ (bottom) animals. **h** Expression of *CSN2* in sorted luminal progenitors from either reduction mammoplasties of healthy controls or prophylactic mastectomies from *BRCA1* carriers. The top panel shows expression in eight controls and eight *BRCA1* carriers of *CSN2* as measured by qPCR. The bottom panel shows expression in four controls vs. four *BRCA1* carriers as measured by RNA-sequencing of sorted luminal progenitors. FC fold change, TF transcription factor, CPM counts per million. Source data for the qPCR is provided as a source data file.

The staging of the premalignant samples allowed us to identify a total of 16 cell types that change in abundance during the early stages of tumourigenesis (false discovery rate (FDR) < 0.1; Fig. 2a, b and Supplementary Fig. 3a, b). We noted a general decrease in various populations of fibroblasts as well as signs of an overt immune reaction characterised by the expansion of myeloid and lymphoid cells. The only epithelial cluster that expanded was a cluster of luminal cells with an expression profile of secretory alveolar cells (Avd) that was virtually absent at Stage 1 and made up more than a third of the epithelium in Stage 4 (Fig. 2a–c and Supplementary Fig. 3a). This cell type also appeared to be the most proliferative in the entire tissue (Supplementary Fig. 3e). Under homoeostasis these cells are restricted to gestational and lactational stages^3^ and arise from hormone-mediated differentiation of luminal progenitors^4^. In fact, despite all animals being nulliparous we observed a progressive differentiation of the luminal progenitor (Lp) compartment towards the alveolar fate (Avd) with Avd accounting for 1.8% (SD = 1.6%) of the epithelium at Stage 1 and 40.4% (SD = 2.3%) at Stage 4 (Fig. 2a, d). This was accompanied by the expression of known markers of alveologenesis such as the milk protein beta-casein (*Csn2*) and the transcription factor *Elf5* (Fig. 2c, d). At the macroscopic level we observed the appearance of what has previously been described as hyper-branching and alveologenesis in a different model of *Brca1/p53*^5^ (Fig. 2e and Supplementary Fig. 3c). We further confirmed the presence of alveolar cells by immunofluorescence, which highlighted the expression of Csn2 at the protein level as well as the presence of alveolar structures (Fig. 2f). Finally, we used assay for transposase-accessible chromatin sequencing (ATAC-Seq) to identify changes in chromatin accessibility of Lps in *Brca1/p53* animals pre-tumour formation (Fig. 2g and Supplementary Fig. 4). We identified increased accessibility at several key genes of alveologenesis such as *Csn2* and *Wap* with proximal enhancer regions known to be more accessible during gestation^6^ (Fig. 2g, highlighted). In addition, chromatin regions with increased accessibility showed significant enrichment for key transcription factors that drive alveolar differentiation including *Cebpb*, *Elf5*, *Nfkb1* and *Sox10* (Fig. 2g and Supplementary Data 1). Together this suggests that luminal progenitors in the *Brca1/p53* mouse model are poised to differentiate towards the alveolar fate and progressively do so during the early stages of tumourigenesis.

Next, we sought to find an indication of whether a similar process might occur in the human breast during tumour development. For this, we performed qPCR to assess *CSN2* expression in FACS-sorted luminal progenitor cells from *BRCA1* carriers who had undergone prophylactic mastectomy (*n* = 8) as well as healthy women undergoing reduction mammoplasty (*n* = 8). We identified two samples from *BRCA1* carriers with noticeably elevated *CSN2* levels and none in the healthy controls (Fig. 2h). To further validate this, we performed RNA-sequencing on an independent set of luminal progenitors from four healthy controls and four *BRCA1* carriers. Again, we found that two out of the four carriers show high levels of *CSN2* (Fig. 2h). Differential expression analysis from those two samples against all other samples showed an enrichment of pathways involved in the recruitment of the immune system as well as positive regulation of *NFKB* (Supplementary Fig. 3d). Although these data lack the cellular and temporal resolution that we have from the mouse model, it does suggest that aberrant differentiation of luminal progenitors also occurs in humans.

**Fig. 3:**
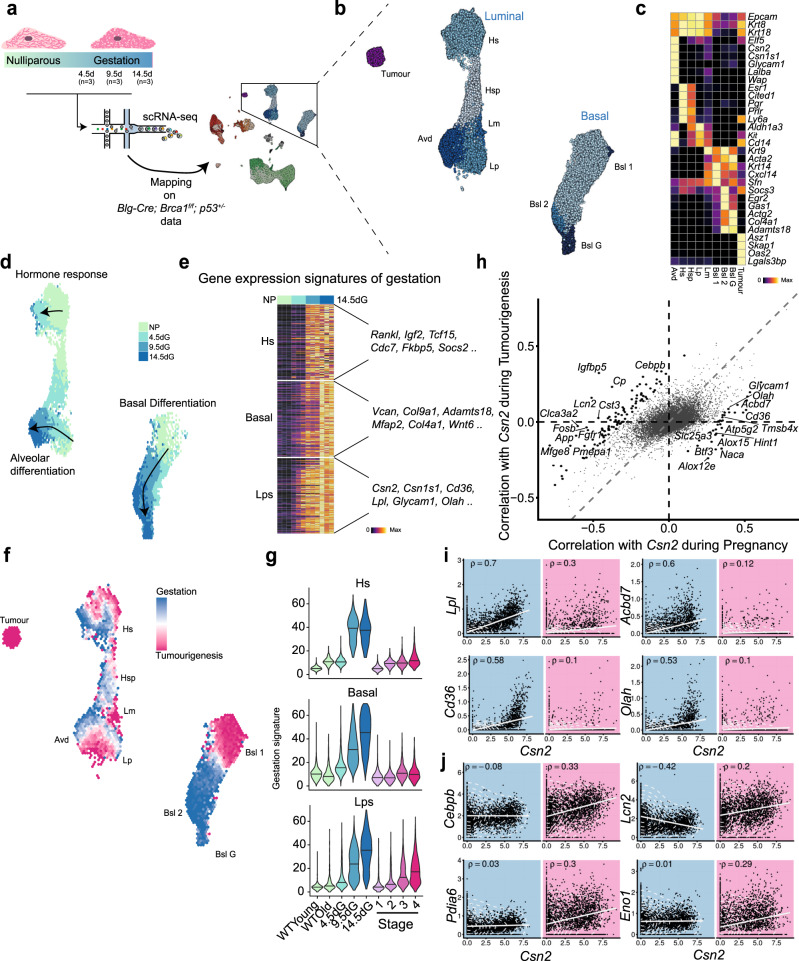
The aberrant differentiation of luminal progenitors in the context of homoeostatic differentiation during gestation. **a** Schematic overview of the experimental strategy. Mammary glands of 12 animals from four time points (Nulliparous, 4.5dG, 9.5dG, 14.5dG; three samples and a minimum of 18,000 cells per time point) were digested to prepare single-cell suspensions for scRNA sequencing after depletion of dead cells. The dataset was integrated with the tumourigenesis dataset presented in Fig. 1. **b** Same UMAP as in **a** showing only the epithelial compartment. **c** Gene expression of marker genes for all epithelial cell types. Values are scaled from 0 to 1 per row. **d** Binned UMAP from **b** only showing cells collected from the gestation time points, coloured by the time point at which the majority of the cells in the respective bin were collected. **e** Gene signatures of gestation for each of the three main epithelial compartments defined as the top 100 up-regulated genes between 14.5dG (Basal and Lps) or 9.5dG (Hs) and nulliparous samples. **f** Binned UMAP from **b** coloured by the percentage of cells in each bin deriving from the tumourigenesis dataset with blue representing 100% of cells deriving from the gestation samples and purple representing 100% of cells derived from the *Blg-Cre; Brca1*^*f/f*^*; p53*^*+/−*^ animals. Datasets were down sampled to the same number of cells. **g** Summed expression of signatures from **e** across all conditions. **h** Differential correlation analysis with *Csn2* during tumourigenesis and gestation computed on all Lps and Avds. The values represent the distance to median correlation in the two conditions. Highlighted dots represent genes with and FDR < 0.001 and |Δ*ρ*|> 0.3. **i**, **j** Some genes from **h** are highlighted. The left (blue) panel represents the correlation with *Csn2* (*X*-axis) during gestation and the right (purple) plot the correlation during tumourigenesis. Gene expression values are normalised, log-transformed counts. The line represents a linear, least-square regression and the dashed lines a 2D density estimate. dG day gestation.

To further characterise the aberrant alveologenesis, we decided to compare it to its homoeostatic counterpart. We performed scRNA-seq on three gestational time points (4.5dG, 9.5dG and 14.5dG) and integrated it with the tumourigenesis data (Fig. 3a–c and Supplementary Figs. 1a and 5). Epithelial maturation during gestation is regulated by systemic hormones, including progesterone released by the corpus luteum^7^. Accordingly, we found transcriptional responses in all epithelial compartments (Fig. 3d, e and Supplementary Fig. 5c). Hormone-sensing cells are known to be the direct responders to pregnancy hormones and in turn release paracrine signalling factors such as *Rankl* (also known as *Tnfsf11)* and *Igf2* (Fig. 3e) to orchestrate the development of the tissue. In response, the basal compartment up-regulates the expression of various collagens and myosins, all of which is required for the contraction of the ducts upon suckling of the infant (Fig. 3e). Finally, we also observe the gradual differentiation of luminal progenitors, which commences at 4.5dG and reaches near-completion at 14.5dG, marked by expression of various milk proteins and genes involved in fatty-acid metabolism (Fig. 3d, e).

Next, we contrasted this molecular reference of gestation with the aberrant phenotype of the *Brca1/p53* animals. We found that hormone-sensing luminal cells in *Brca1/p53* animals lack the transcriptional response observed during pregnancy, indicative of an absence of progesterone signalling (Fig. 3f, g). This is corroborated by the absence of basal differentiation during *Brca1-*mediated tumourigenesis, indicating that the gestation-like phenotype is hormone-independent, and a cell-autonomous process restricted to the luminal progenitor compartment (Fig. 3f, g).

To directly compare the alveolar differentiation between gestation and early steps of tumourigenesis we identified genes that differ in their correlation to *Csn2*. This analysis revealed 137 genes with a differential correlation (FDR < 0.001 and |Δ*ρ*| > 0.3, Fig. 3h). For example, during tumourigenesis we observed no correlation between *Csn2* expression and numerous genes involved in fatty-acid metabolism which are normally induced during gestation (Fig. 3i). This suggests that the alveolar cells found during early stages of tumourigenesis are unlikely to be fully functional, secretory cells. Genes that showed a positive correlation with *Csn2* only during tumourigenesis included a number of factors that are associated with basal-like breast cancer, among them a master regulator of alveologenesis *Cebpb*^8^ (Fig. 3j and Supplementary Fig. 5d). Interestingly, multiple studies have shown that *Cebpb* as well as other regulators of alveologensis such as *Nfkb1* can be induced in response to DNA damage^9,10^. Therefore, this response could unintentionally drive a transcriptional program of alveolar differentiation in this setting, which is supported by the enrichment of *Cebpb* and *Nfkb1* binding sites in accessible chromatin of luminal progenitors (Fig. 2g).

**Fig. 4:**
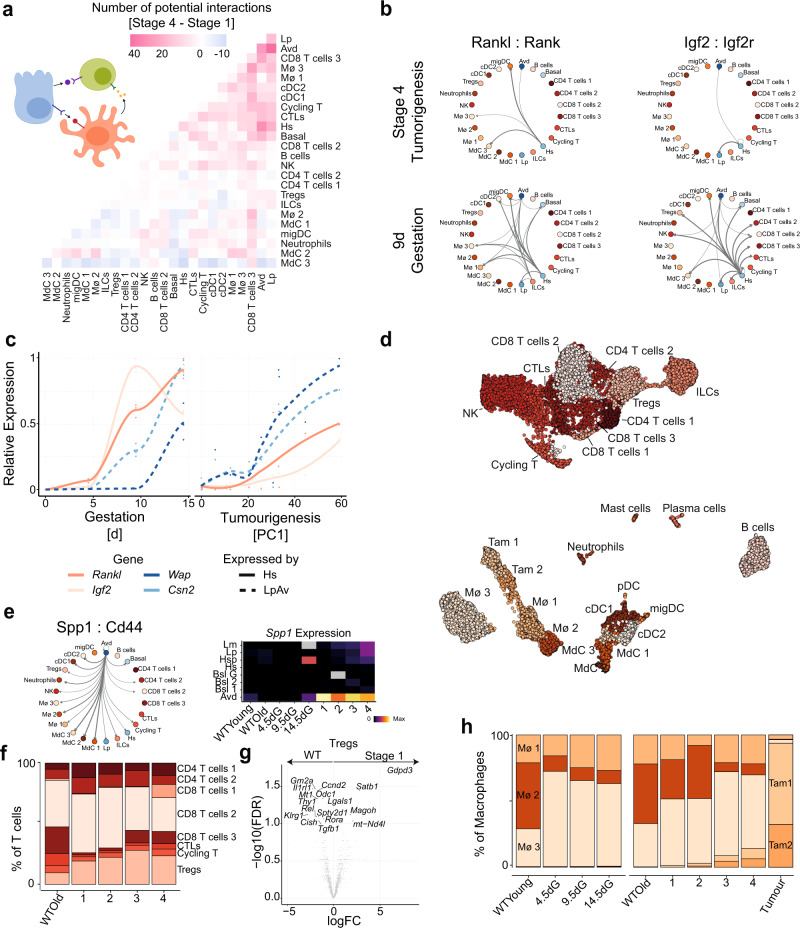
Aberrant differentiation of luminal progenitor cells is accompanied by an altered microenvironment with tumour-promoting characteristics. **a** Net difference in the number of potential interactions between any immune and epithelial cell types between stage 4 and stage 1. The number of potential interactions was estimated in each stage using cellphoneDB at an FDR of 0.05. **b** Graphs representing potential interactions for Rankl:Rank and Igf2:Igf2r for Stage 4 of tumourigenesis (top row) and 9dG (bottom row). Nodes represent cell types and edges represent significant interactions with the width of the edge illustrating the mean expression of ligand and receptor. The arrow of the edges represents the direction from ligand expressing to receptor expressing. **c** Gene expression for the paracrine signalling factors *Rankl* and *Igf2* in hormone-sensing cells and the alveolar markers *Csn2* and *Wap* in luminal progenitors and alveolar cells. Expression is scaled across gestation and tumourigenesis to 0 and 1. In the tumourigenesis panel, the *X*-axis represents the values of PC1 that were scaled by (PC1 + min(PC1))/max(PC1−min(PC1)) × 100 **d** UMAP for all immune cell types captured in the gestation and tumourigenesis dataset. **e** Interaction plot as in **b** for Spp1:Cd44 during stage 1 of tumourigenesis. Right panel shows mean log expression of *Spp1* across epithelial cell types in the mammary gland across various conditions. Grey represents conditions with no cells of that particular cell type. **f** Barplot of relative frequency of T-lymphocytes during tumourigenesis. **g** Differential expression analysis of Tregs from old wild-type animals and Tregs from stage 1. **h** Distribution of macrophage populations during gestation and tumourigenesis as in **g**. Data in barplots represent the mean per stage. For all pregnancy time points *n* = 3 independent animals were analysed; for the tumourigenesis stages the sample sizes are specified in Supplementary Fig. 1a. FC fold change, FDR false discovery rate.

The analysis so far suggests that the early stages of TNBC development in the *Brca1/p53* model are primarily characterised by the cell-autonomous differentiation of the luminal progenitor compartment. To further understand how this affects the composition of surrounding cells (Fig. 2a, b), we identified potential cell–cell communication pathways using CellPhoneDB^11^, a database of curated ligand receptor pairs associated with a statistical framework to test for enrichment of signalling pathways between cell types in scRNA-seq data. When computing the difference in the number of potential signalling axes among the various epithelial and immune cells found in stage 1 and stage 4, we find an increase in heterotypic signalling, clustering around the luminal progenitors and alveolar cells (Fig. 4a). For example, we see that later stages show a signalling axis from hormone-sensing cells to developing alveolar cells via Rankl:Rank and Igf2:Igf2R both of which are known to induce alveologenesis^12^ (Fig. 4b and Supplementary Fig. 6a). This is in line with previous data that highlighted a dysregulation of *RANKL* in *BRCA1* carriers^13^. In contrast to normal development, however, we find that aberrant differentiation precedes Rankl expression from hormone-sensing cells (Fig. 4c and Supplementary Fig. 6c), suggesting that induction of *Rankl* expression is a means to further potentiate the aberrant differentiation. We note that there are several potential signalling axes from alveolar cells to hormone-sensing cells including Fgf1 and Lif, both of which have been shown to induce Rankl expression^14^ (Supplementary Fig. 6b).

The analysis also revealed an increase in the number of potential signalling axes between the epithelium and some cells of the immune system (Fig. 4a, d and Supplementary Fig. 6d). For example, we found a potential interaction between osteopontin (Spp1) expressed by Avd with Cd44 expressed on immune cells across all stages (Fig. 4e). *Spp1* is up-regulated specifically in Avd during tumourigenesis and ultimately also highly expressed by the tumour (Fig. 4e and Supplementary Fig. 6e). Previous research suggests that the Spp1:Cd44 signalling axis acts as an immune checkpoint thus, inducing host tolerance during tumour formation^15^. Additionally, we find an expansion of Tregs suggesting the early establishment of an immuno-suppressive environment (Fig. 4f, Fig. 2b). Compared to wild-type animals, Tregs from stage 1 show reduced expression of *Klrg1* and *Il1rl1*, two markers of tissue resident Tregs, suggesting an early influx of Tregs from the circulatory system preceding tumour formation (Fig. 4g).

In the myeloid compartment we identified three types of tissue resident macrophages (Mø 1–3) matching the recent classifications in the field^16,17^. In line with Dawson et al. we found the alveolar-associated macrophages Mø 3 to be the dominating macrophage phenotype during gestation (Fig. 4h). Interestingly, we find a similar expansion of Mø 3 during the premalignant stages of tumourigenesis (Fig. 4h). As this subtype has been shown to be required for tissue remodelling it most likely fulfils a similar role in the context of tumour development, supported by a relative enrichment for the expression of genes with metalloendopeptidase and collagen binding activity (Supplementary Fig. 7). We further found two types of tumour-associated macrophages Tam 1 (marked by *Arg1*, *Spp1* and *Trem2*) and Tam 2 (marked by *C1qb*, *C1qc*, *Lgmn* and *Apoe*) (Supplementary Fig. 7)^18^. These seem to be recruited already in stage 3 and 4 before a macroscopic tumour is visible, potentially establishing an immuno-suppressive environment early on.

## Discussion

One of the major hurdles for the early detection of cancer is our poor understanding of tumour-initiating events. In humans it is challenging to assess the immediate impact of genetic alterations on the cellular dynamics of the tissue. Here we demonstrate the utility of time-resolved single-cell profiling of genetically engineered mouse models before tumour formation to address this challenge. We found that perturbing *Brca1/p53* in the putative cell of origin, luminal progenitors^1,2^, induces an aberrant alveolar differentiation pre-malignancy. Unlike the hormonally driven alveolar differentiation that occurs during gestation, this process is cell autonomous and characterised by the dysregulation of transcriptional regulators of alveologenesis. Based on our data we propose a model where transcriptional and epigenetic changes driven by *Brca1/p53* inadvertently promote a differentiation program hardwired in luminal progenitors, highlighting the deterministic role of the cell of origin and offering a potential explanation for the tissue specificity of *BRCA1* tumours. Despite the dense, longitudinal sampling it remains unclear at which point in the herein described differentiation trajectory the first tumour cells emerge, and at which point they should be denoted as such. We do note, however, that the tumours in our study as well as human TNBCs express transcriptional regulators of alveologenesis such as *Elf5*, *Sox10*, *Foxc1* and *Cebpb* (Supplementary Figs. 5 and 10). Yet, inferring the cellular lineage of the tumour precisely will require advanced lineage tracing studies. Our experimental approach has allowed us to further identify responses in the surrounding cellular compartments during the early steps of tumourigenesis. In particular, we highlight the establishment of a potentially immuno-suppressive environment pre-malignancy marked by the recruitment of Tregs and tumour-associated macrophages. Finally, this dataset can also be used as a resource for the community to understand the relationship between the developing tumour and other stromal compartments. In addition, we also show that aberrant differentiation is detectable in some human *BRCA1* carriers. With the advent of spatial transcriptomics, it will be interesting to investigate the potential spatial dynamic of this aberrant differentiation process in *BRCA1* carriers. Future efforts should investigate the efficacy of detectable aberrant differentiation and the accompanied changes in the microenvironment in stratifying women at high risk of TNBC in the clinic, thus potentially reducing unnecessary invasive screening and surgical interventions.

## Methods

### Mouse experiments

All experimental animal work was performed in accordance to the Animals (Scientific Procedures) Act 1986, UK and approved by the Ethics Committee at the Sanger Institute. The *Blg-Cre;Brca1*^*f/f*^*;p53*^*+/−*^ (JAX 012620)^19^ mouse model was used to study TNBC tumour development. In detail, tissues were collected from 13 nulliparous mice with age ranging from 30 to 48 weeks (Supplementary Fig. 1a). At time of collection, 11 mice showed no presence of tumours, while 2 presented tumours in one of the glands. In addition, we collected glands from two *Blg-Cre;Brca1*^*f/f*^*;p53*^*+/+*^ that were used as validation for the ordering of the samples (Supplementary Fig. 2d). For the tumour-bearing mice, contralateral glands and tumours cleared of surrounding mammary gland tissue were treated as independent samples in the dataset. For the pregnancy time points, females were mated with studs. Tissues were then harvested from three individual mice per time point at gestation day 4.5, 9.5, and 14.5. Tissue from nulliparous wild-type females was harvested at 12 weeks of age for comparison to the pregnancy time points (young nulliparous, *n* = 3), and at 53 and 74 weeks of age for comparison to the premalignant and tumour stages (old nulliparous). For the ATAC-Seq experiment, two wild-type and two *Blg-Cre;Brca1*^*f/f*^*;p53*^*+/−*^ mice (aged between 36 and 40 weeks) were used. All mice were housed in individually ventilated cages under a 12:12 h light–dark cycle, with water and food available ad libitum and euthanized by terminal anaesthesia. All the primers used for genotyping are listed in Supplementary Data 2.

### Human tissues

All primary human breast tissue was derived from women undergoing reduction mammoplasties with no known genetic history (*n* = 12) and prophylactic mastectomies from women with germline *BRCA1* mutations (*n* = 12, one of which had a tumour in the contralateral gland) under full informed consent either at Addenbrooke’s Hospital, Cambridge, UK, in accordance with the National Research Ethics Service, Cambridgeshire 2 Research Ethics Committee approval (08/H0308/178) as part of the Adult Breast Stem Cell Study or obtained from the Breast Cancer Now Tissue bank, as approved by Cambridge Central REC (15/EE/0192). (Supplementary Data 3).

### Mammary gland dissociation into single-cell suspension

Lymph node divested mouse mammary glands (excluding the cervical pair) were mechanically dissociated after collection, pooled per animal and the finely minced tissue was transferred to DMEM/F12 (Gibco) + 10 mM HEPES (Gibco) + 2 mg ml^−1^ collagenase (Roche) + 200 U ml^−1^ hyaluronidase (Sigma) (CH) + gentamicin (Gibco) at 37 °C and vortexed every 30 min. After the lysis of red blood cells in NH_4_Cl, cells were briefly digested with warm 0.05% Trypsin-EDTA (Gibco), 5 mg ml^−1^ dispase (Sigma) and 1 mg ml^−1^ DNase (Sigma) and filtered through a cell strainer (BD Biosciences).

Frozen vials of human epithelial-enriched fractions obtained from the Cambridge Breast Cancer Unit and dissociated as in ref. ^20^ or of organoids from the Breast Cancer Now tissue bank were defrosted and diluted in cold HBSS 1% FCS (HF), further digested with warm Trypsin-EDTA (Gibco), 5 mg ml^−1^ dispase (Sigma) and 1 mg ml^−1^ DNase (Sigma) and filtered through a 40μM cell strainer (BD Biosciences).

### Cell labelling followed by flow cytometry and sorting

Mouse and human mammary cells were incubated in HF medium (Hank’s balanced salt solution (Gibco) + 1% fetal bovine serum, Gibco) + 10% normal rat serum (Sigma) for 20 min on ice to pre-block. Mouse mammary cells were stained with the following primary antibodies: Cd31-biotin (eBioscience, clone 390, 1 µg ml^−1^, 1:500); Cd45-biotin (eBioscience, clone 30F11, 1 µg ml^−1^, 1:500); Ter119-biotin (eBioscience, clone Ter119, 1 µg ml^−1^, 1:500); EpCAM-APC/Cy7 (Biolegend, clone G8.8, 0.5 µg ml^−1^, 1:500); Cd49f-BV421 (Biolegend 313623, 2 µg ml^−1^, 1:100); Cd49b-AF488 (Biolegend, clone HMα2, 1 µg ml^−1^, 1:500) and Sca1-AF647 (Biolegend, clone D7, 1 µg ml^−1^, 1:500). Cells were then stained with Streptavidin-PE/Cy7 (BD Biosciences, 0.4 µg ml^−1^, 1:500). Zombie Aqua (Biolegend, 1:100) was used to detect dead cells. Human mammary cells were stained with the following primary antibodies: CD45-APC (Biolegend, clone H130,1:100), CD31-APC (Biolegend, clone WM-59, 1:100), EPCAM-APC/Fire750 (Biolegend, clone 9C4, 1:50), CD49f-PE/Cy7 (Biolegend, clone GoH3, 1 µg ml^−1^, 1:200). DAPI was used to detect dead cells. Cells were filtered through a cell strainer (Partec) before sorting. Sorting of cells was done using a FACS Aria Fusion sorter. Single-stained control cells were used to perform compensation manually. Unstained cells were used to set gates. After doublets, dead cells and contaminating haematopoietic, endothelial and stromal cells were gated out, human luminal progenitors were sorted for RNA processing and mouse CD49b^+^, Sca1^−^ luminal progenitors were sorted for ATAC-Seq experiments. The gating strategies are reported in Supplementary Figs. 8 and 9.

### scRNA sequencing of mouse samples

MACS Dead Cell Removal kit was used to exclude dead cells from single-cell suspensions. Subsequently, cells were spun down and resuspended in HF. Samples were manually counted using an improved Neubauer chamber and the cell concentration was normalised by addition of HF. Equal numbers of cells per sample were processed for scRNA library preparation. Samples were processed for first-strand cDNA synthesis within 6 h from tissue isolation. The remaining steps of library preparation were completed within the following 7 days.

### Whole mounts

For whole mount analysis, n. 4 abdominal glands were spread out using forceps on a glass slide and incubated in Carnoy’s fixative overnight. The slide was then placed in carmine alum (Sigma) stain overnight. The slide was returned to Carnoys and imaged using a Leica MZ75 dissecting microscope.

### Immunofluorescence

Five micrometer sections of mammary glands were immunostained with antibodies for Csn2 (sc-166530, Santacruz, 1:50) and K8 (TROMA-1, MABT329, Merk-Millipore, 1:500). Secondary staining involved goat anti-rat AlexaFluor 647, or anti-mouse AlexaFluor 594 (1:200, Invitrogen). Nuclear stain was detected using ProLong Gold Antifade Mountant with DAPI (Thermofisher, P36941).

### Confocal microscopy and image analysis

Immunofluorescence images were acquired using a Leica TCS SP5 inverted confocal microscopes with ×40/1.3 HC PL APO objective lens. Laser power, line averaging and step increment were adjusted manually to give optimal fluorescence intensity for each fluorophore with minimal photobleaching.

### Library preparation and sequencing

Library preparation of murine samples was performed according to instruction in the 10X Chromium single cell kit v2 (Batch 1 and 2) or v3 (Batch 3–5). The samples were processed in five batches (Supplementary Figs. 1a and 5a) where each batch represents a day in which multiple biological samples (one biological sample represents either pooled glands from one mouse or a tumour from one mouse) were processed together. The libraries were then pooled and sequenced on a HiSeq4000 (PE26/98) or NovaSeq6000 (PE28/91).

### Processing and quality control of scRNA-seq data

Read processing was performed using the 10X Genomics workflow. Briefly, the Cell Ranger Single-Cell Software Suite (3.10) was used for demultiplexing, barcode assignment and UMI quantification (http://software.10xgenomics.com/single-cell/overview/welcome). The reads were aligned to the pre-built mm10 reference genome provided by 10x Genomics (https://support.10xgenomics.com/single-cell-gene-expression/software/downloads/latest).

Samples from the tumourigenesis dataset and the pregnancy dataset were processed independently to generate two high-quality, filtered data sets prior to merging. The former consisted of three batches and the latter of two. All steps below were performed individually for the two data sets, when results or settings are presented the values in parentheses represent the results or settings for the pregnancy data.

Barcodes that correspond to droplets with successfully captured cells were distinguished from empty droplets using the “emptyDrops” function from DropletUtils^22^ at an FDR of 0.01. We then used the following metrics to flag poor-quality cells or outliers: number of genes detected, total number of unique molecular identifiers (UMIs), percentage of molecules mapped to mitochondrial genes as well as the detection trend (see below). Cells with a number of genes detected and total number of UMIs that was greater or smaller than median ± 3 × MAD (median absolute deviation) or a percentage of molecules mapped to mitochondrial genes greater than median + 5 × MAD were then excluded from the downstream analysis. The detection trend was defined as a cubic spline regression of genes detected on the number of UMIs sequenced in log space. Cells with a residual smaller than median −6 × MAD were identified as outliers, most of which were red blood cells (RBCs). This resulted in a total of 124,507 (102,829) cells being considered for further analysis. Gene expression values were then normalised per-batch by size factors that were estimated using the “computeSumFactors” function in scran before being scaled across batches using “muliBatchNorm”^23,24^.

### Highly variable genes

Highly variable genes (HVGs) were identified by first fitting a mean-dependent trend to the gene-specific variances to all genes assuming that this trend is dominated by technical variance. This trend was then defined as the technical component of the variance. The genes with a positive residual variance were defined as HVGs or a fraction thereof if computational speed was a priority, e.g. for doublet detection. From the list of HVGs we excluded all genes that were annotated as constituents of the ribosome (GO:0003735, GO:0005840, GO:0015935, GO:0015934) or encoded by the mitochondrial genome as these tend to be driven by technical variation.

### Doublet detection and data filtering

Due to the high number of cells and samples, droplets with multiple cells are particularly problematic as they will be captured in a sufficient number to form distinct clusters. We therefore tried to identify doublets before clustering and annotating the data. We used relatively liberal thresholds to avoid erroneously removing cells. Briefly, the probability of being a doublet was estimated for each cell per sample (that is one 10× lane) using the “doubletCells” function in scran^23^ using only HVGs. Next, we used “cluster_walktrap”^25^ on the SNN-Graph that was computed on HVGs to form highly resolved clusters per sample. Per-sample clusters with >median  + 1.5 × MAD) doublet score that made up less than 5% of the sample were tagged as doublets. This was followed by a second round of per-dataset clustering, in which again cells belonging to clusters with a high proportion (>2 × MAD from median) of cells previously labelled as doublets were also defined as doublets. At this point we also excluded clusters with a non-zero median expression of haemoglobins as these represent contaminating RBCs. Clusters most likely representing stripped nuclei as defined as clusters with less than a 0.005 median fraction of mitochondrial reads were also excluded^26^. In total, this led to the further exclusion of 2439 (3047) cells.

### Batch correction

To account for technical differences between experimental batches we matched mutual nearest neighbours across batches^27^. This step was performed both within the data sets before and after doublet removal as well as across the datasets to integrate the pregnancy and tumourigenesis data (Fig. 3a). For this we applied the “fastMNN” function from batchelor with “k = 20”, “cos.norm.out=FALSE” and d = 50 on the normalised gene expression values of HVGs. For HVG detection, the variance was decomposed per-batch as described above and then combined using the “combineVar” function in scran;^23^ all genes with a positive residual variance were then defined as HVG. Visual inspection of the data after batch correction suggested that most of the effect was removed and that the biological signal now dominates the structure (Supplementary Fig. 5a). The batch-corrected principal components were used for dimensionality reduction and clustering. All differential expression tests were performed on non-corrected, normalised gene-expression values with an added blocking factor for batch.

### Dimensionality reduction

All two-dimensional representations of the scRNA-seq data were computed using UMAP (Uniform Manifold Approximation and Projection for Dimension Reduction)^28^. The UMAP coordinates were computed based on the batch-corrected principal components using the umap function from the umap package with default settings and “random_state=42”. For ease of interpretation, all UMAP embeddings represent the coordinates computed on the integrated dataset, that is pregnancy and tumourigenesis. In Figs. 1 and 2 only samples of the tumourigenesis dataset are shown, whereas in Figs. 3 and 4 cells from all samples are shown. The gene expression plots in Fig. 2 as well as the UMAPs coloured by time point and condition (Fig. 3) were produced by binning cells into hexagonal bins using the schex package (Freytag S (2020). schex: Hexbin plots for single cell omics data. R package version 1.2.0, https://github.com/SaskiaFreytag/schex).

### Clustering and cell type annotation

The data were clustered first individually per dataset including a preliminary annotation. This annotation was mainly used to identify clusters that represent remaining doublets or damaged cells which allows removal before the final integration step of the two datasets.

Clustering was performed using the walktrap algorithm on the Graph from the UMAP embedding using the cluster_walktrap function in igraph with “step=7” (6 for the tumourigenesis data, 7 for pregnancy)^25^. Before annotating cell types, we performed a post-hoc test by iteratively merging clusters with <10 differentially expressed genes (FDR < 0.1 and minimum log fold change of 1) using “findMarkers” from scran. Ribosomal genes and mitochondrial genes were excluded at this stage for differential expression (DE) analysis (see above). Some clusters were manually sub-clustered if there was structure apparent based on gene expression of common marker genes or as observed in the UMAP embedding. The sub-clustering was performed on an SNN-Graph as computed on the batch-corrected principal components using either louvain or walktrap clustering. Finally, remaining clusters that had <10 DE genes as defined by “findMarkers” were merged to their closest cluster. The exception to this approach were two superclusters of T-Cells that represented known biological subtypes with little DE one consisting of CD4, Tregs and CD8 T-Cells the other containing CTLs, NK and CD8 cells. In this case the clusters were kept separate despite showing less than 10 DE genes.

### Transcriptional ordering of samples

Despite the age of the animals being highly correlated with the underlying biological process of tumour development it does not directly represent the stage of disease development. This is largely due to the stochasticity of the many processes involved in tumour development including but not limited to the acquisition of further mutations upon the loss of *Brca1* and *p53*. The approach that was used in this study is based on the assumption that there are stereotypical processes in the transcriptomes of the captured cells that represent the biological process of tumour formation, including, for example, a response of the immune system. The latent factor most likely representing biological time was identified using PCA computed on the pseudo-bulked and TMM normalised, log-transformed counts. We interpreted the first principal component to represent tumourigenesis based on the high correlation with age (Supplementary Fig. 2b), the separation of tumours from mammary gland samples (Supplementary Fig. 2a), and the enrichment of genes in processes such as immune response and cell cycle progression. We defined PC1 as –1 × PC1 in order to have WT samples on the left of the PCA and tumours on the right, this is a purely aesthetic change and has no other impact. Further, we projected two independently collected samples from 42 week old *Blg-Cre, Brca1*^*f/f*^*;p53*^*+/+*^ animals, which also develop TNBC albeit with much longer latency^19^, onto the PCA space (Supplementary Fig. 2d). These samples received low PC1 values and were substantially older than other mice in the same bin, supporting the notion that PC1 represents tumour formation and that this is delayed in *Blg-Cre, Brca1*^*f/f*^*;p53*^*+/+*^ animals.

### Differential abundance testing

To identify changes in cell-type-specific abundance during the premalignant stages we regressed the scaled PC1 values on the normalised log counts for each cell type using robust regression as implemented in the “rlm” function of the MASS package^21^ with default settings and “max_it=100”. Normalised log counts of cluster abundance were computed using the “cpm” function in edgeR accounting for total number of cells per sample. To assess statistical significance of the regression we employed a robust *F*-test as implemented in sfsmisc. The resulting *P* values were corrected for multiple testing using the Benjamini–Hochberg method. Prior to fitting, the PC1 values of all samples were scaled so that the sample with the smallest PC1 value is set to 0 and the one with the highest PC1 value a 1. This way the estimated coefficient (logFC) is interpretable and represents the estimated average change in abundance of a particular cell type from the first to last sample. This was performed on all samples from stage 1 to 4 and clusters with more than an average of 10 cells per sample.

### Differential correlation analysis

In order to identify genes that are differentially regulated during gestation and tumourigenesis, we tested for genes that are differentially correlated with Csn2 in the two conditions using the scHOT approach^29^. This was performed on all cells belonging to the Lp and Avd cluster using Spearman correlation testing for genes with at least 10 non-zero observations in both groups.

### Cell–cell interactions

Potential cell–cell interactions were identified using cellphoneDB^11^. This was performed on all epithelial and immune cell types that were present in all conditions excluding the tumour samples. Further the basal clusters Bsl 1, Bsl 2 and Bsl G were grouped into “Basal” because Bsl 2 and Bsl G contained only a small number of cells in the tumourigenesis dataset and Hsp and Hs were combined into Hs. The mouse ENSEMBL IDs were mapped to the human orthologoues as defined in the ENSEMBL database accessed via the biomaRt package. For the visualisation of specific interactions we computed a directed graph where each node represent a cell type and each edge a significant interaction with the weight of the interaction representing the mean expression of ligand and receptor. This is based on the visualisation proposed in the comunet package^30^.

### Differential expression

Differential gene expression analysis was performed using edgeR^31^. A negative binomial generalised log-linear model was fitted to the remaining genes with the cluster assignments as covariate(s). The “glmQLFTest” function was used to identify genes that have LFC significantly different from 0 at an FDR of 0.1. The marker genes used for cell type inferences were identified using the “findMarkers” function in scran with default settings.

### Gene ontology enrichment analysis

A gene set enrichment analysis based on gene ontology (GO) terms was conducted to characterise various genesets in the analysis. The genes of interest were compared to all genes that were tested using topGO^32^.

### ATAC-Seq

Using the previously established ATAC-sequencing protocol^33^ the tagmentation reaction was performed on FACS-sorted luminal progenitors isolated from nulliparous mice either wild-type (*n* = 2, age 40 weeks) or *Brca1/p53* (*n* = 2, age 36 and 38 weeks). Library preparation was performed by the NGS Facility at the Wellcome Trust Medical Research Council Stem Cell Institute using the Nextera DNA Library Prep Reference Guide. Resulting libraries were pooled across all samples and sequenced across one lane of the Novaseq6000.

Resulting reads were subject to quality processing by trimming off the adapter sequences using TrimGalore in paired-end mode with default error rate,–nextera option for transposase sequence filtering and excluding reads with Phred score below 30. Forward and reverse reads were subsequently aligned to the mm10 genome using the BWA-MEM algorithm^34^. Mitochondrial reads were removed using SAMtools. PCR duplicates were marked with MarkDuplicates from Picard tools. Reads shorter than 30 bp were discarded with alignmentSieve from deepTools^35^. Using SAMtools view, reads were quality filtered leaving only unique, mate-mapped reads and removing chimeric alignment and Picard marked PCR duplicates.

Coverage tracks were generated from quality processed BAM files using bamCoverage from deepTools with the counts per million normalisation and 10-base pair long bins. The resulting bigwig files with normalised counts were visualised using the Integrative Genomics Viewer (IGV)^36^.

Differentially accessible sites between wild-type and Brca1/p53 luminal progenitor cells were identified using the csaw package^37^ in R. After loading the QC-filtered BAM file, the ENCODE blacklisted regions were discarded and reads subsequently counted in windows of fixed genomic intervals (20 bp). Low count windows were filtered using the global enrichment approach with 10,000 bp bin size and keeping windows that are threefold different from the background estimate. Normalisation factors were calculated from high abundance windows to eliminate efficiency bias. Differentially accessible sites were identified using edgeR^31^ with FDR < 0.1. Enriched motifs in the resulting differentially accessible genomic regions were found using the findMotifsGenome.pl script from HOMER^38^ using the size-given option to include the exact size of each differentially accessible site.

### Preparation of RNA for qPCR

Sorted cells were spun down and resuspended in RLT, and RNA was extracted using the RNeasy mini kit (for mouse cells) or the RNeasy micro kit (for human cells; Qiagen) according to manufacturer’s instructions. DNA was degraded by adding 20U Rnase-free DnaseI (Roche) for 30 min at room temperature. DnaseI treatment was performed on columns.

### Preparation of cDNA and qPCR

Total RNA was diluted to a final volume of 11 µl. Two microliters of random primers (Promega) were added after which the mixture was incubated at 65 °C for 5 min. A master mix containing Transcriptor Reverse Transcriptase (Roche), Reverse Transcriptase buffer, 2 mM dNTP mix and RNasin Ribonuclease Inhibitors (Promega) was then added. This mixture was incubated at 25 °C for 10 min, then 42 °C for 40 min and finally 70 °C for 10 min. The resulting cDNA was then diluted 1:2.5 in H_2_O for subsequent use. qPCR was performed using a Step-One Plus Real-Time PCR System (Thermofisher Scientific). Taqman (ThermoFisher Scientific) probes with GoTaq Real Time qPCR Master Mix (Promega) were used. The enrichment was normalised with control mRNA levels of *GAPDH* and relative mRNA levels were calculated using the ΔΔCt method compared to the control group. For the list of probes see Supplementary Data 2.

### Reporting summary

Further information on research design is available in the Nature Research Reporting Summary linked to this article.

## Supplementary information

Supplementary Information

Description of Additional Supplementary Files

Supplementary Data 1

Supplementary Data 2

Supplementary Data 3

Reporting Summary

## Data Availability

The authors declare that all data supporting the findings of this study and unprocessed images are available within the article and its supplementary information files or from the corresponding author upon reasonable request. The raw sequencing data are available on ArrayExpress with the following accession numbers: E-MTAB-10043 (scRNA-Seq), E-MTAB-10046 (RNA-Seq) and E-MTAB-10054 (ATAC-Seq). Processed data can also be explored and downloaded at http://marionilab.cruk.cam.ac.uk/BRCA1Tumourigenesis. All computational analyses were performed in R (Version 3.4.1) using standard functions unless otherwise indicated. All code is available online at https://github.com/MarioniLab/Tumorigenesis2018. Source data are provided with this paper.

## Source data

Source Data
