# Supplementary material for: Time-resolved single-cell analysis of Brca1 associated mammary tumourigenesis reveals aberrant differentiation of luminal progenitors
Source: Nat Commun. 2021 Mar 9;12:1502. doi: 10.1038/s41467-021-21783-3 (PMC7940427; doi:10.1038/s41467-021-21783-3)
Supplement: Supplementary file 1 — Supplementary Information [file 41467_2021_21783_MOESM1_ESM.pdf]

Supplementary Figure 1

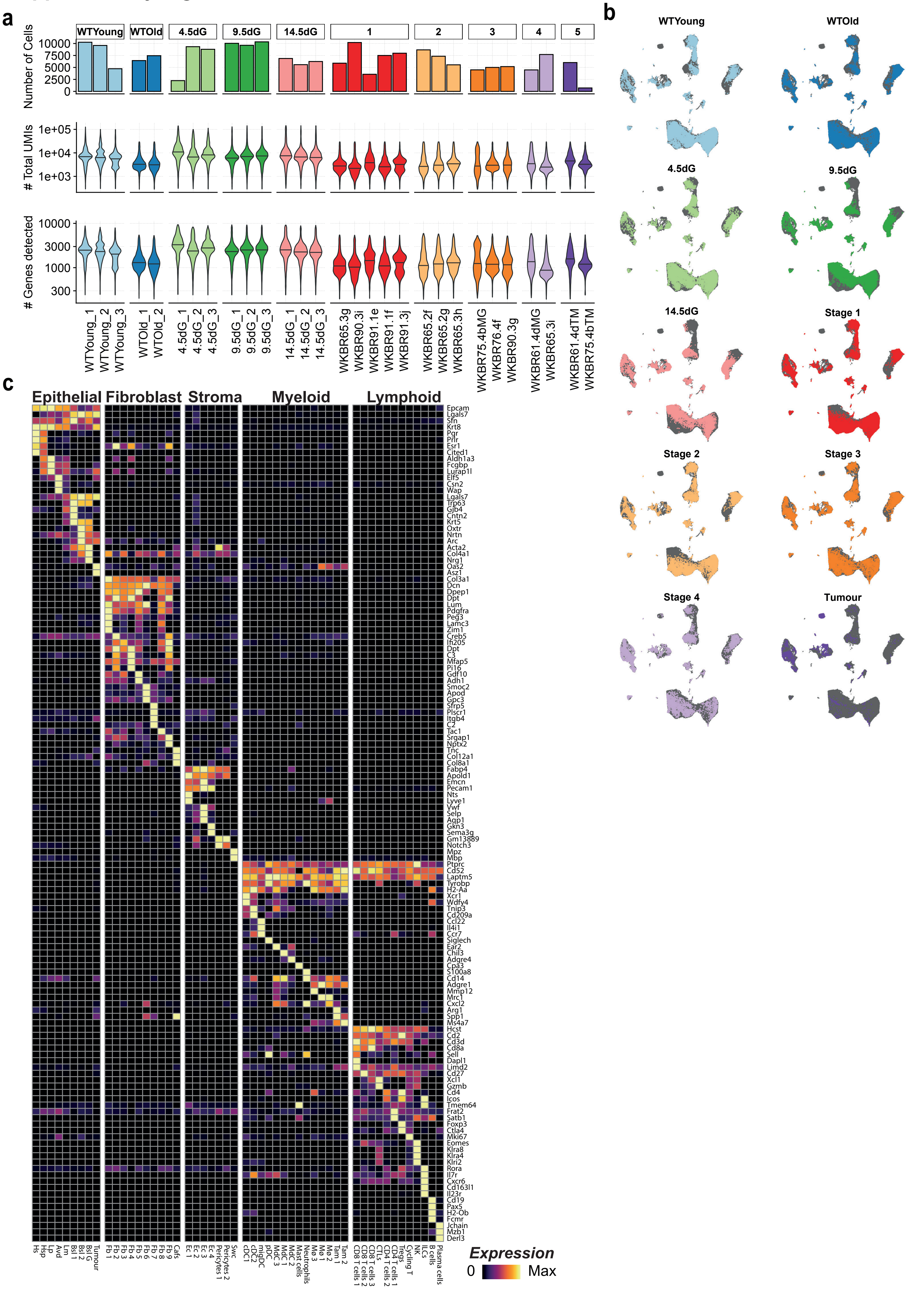

Supplementary Figure 1 Quality control and cell type annotation of scRNA-seq used in the study. (a) QC parameters and number of cells for each of the individual scRNA-seq samples including the gestation data from Figure 3. The violin plots are scaled by width and the line represents the median. **(b)** Similar to Figure 1C UMAP facetted by the different conditions. Coloured by condition. **(c)** Expression of marker genes that were used during cell type annotation.

Supplementary Figure 2

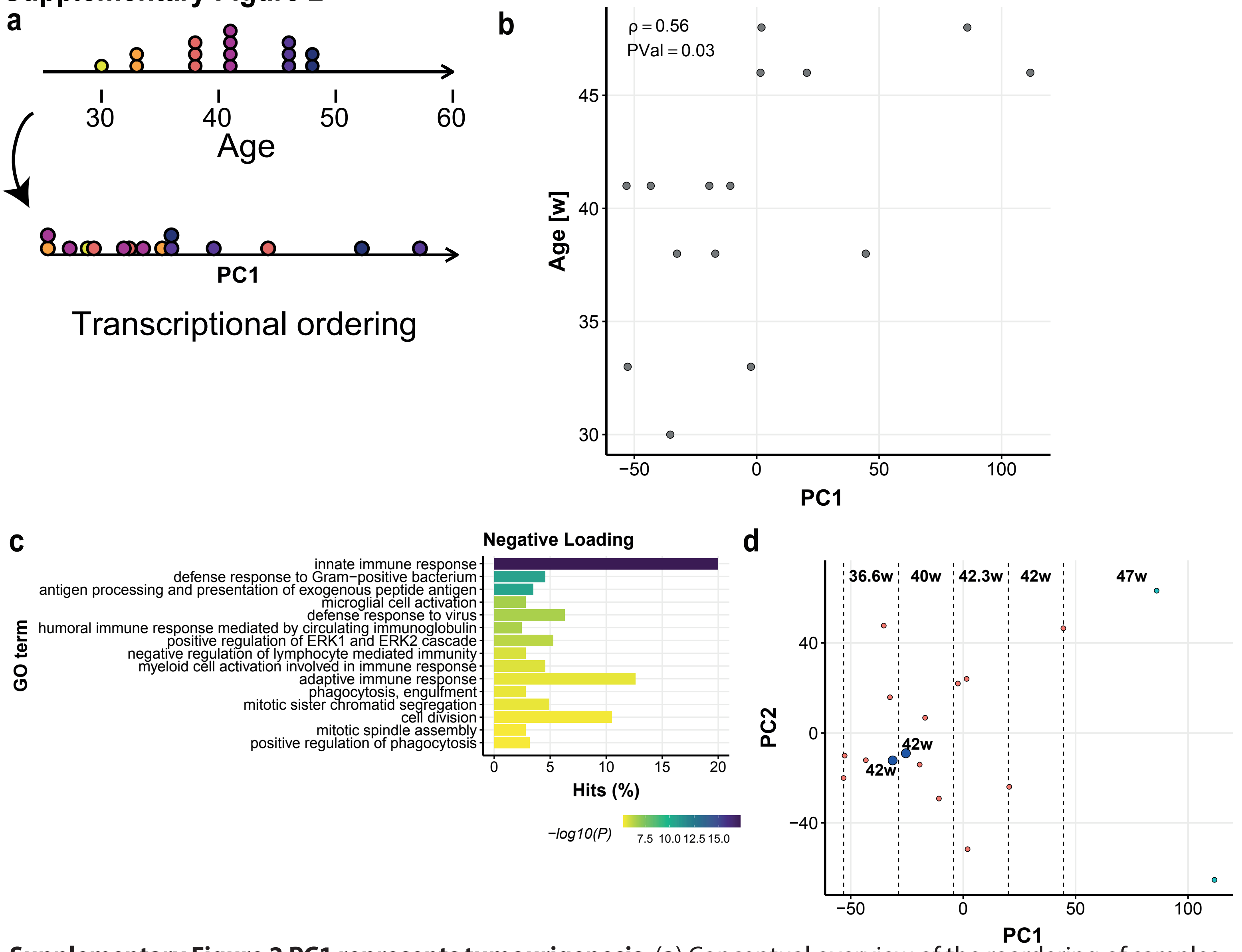

Supplementary Figure 2 PC1 represents tumourigenesis. (**a**) Conceptual overview of the reordering of samples. Top panel depicts biological age of the sample with each dot representing one sample coloured by age. Bottom panel shows distribution of samples along PC1 coloured by age; the x-axis represents the process of tumourigenesis. (**b**) Correlation of age of the samples with PC1. Correlation represents the pearson moment correlation coefficient and the P value is derived from a two-sided test. (**c**) Gene set enrichment analysis for GO-Terms of genes with a negative loading below –0.02. (**d**) Projection of pseudo-bulked scRNA-seq samples from two 42-week-old *Blg-Cre; Brca1*$^{f/f}$*; p53*$^{+/+}$ animals, that also develop TNBC albeit with much longer latency[19] onto the PCA space from Figure 1b. These samples received low PC1 values and were substantially older than other mice in the same bin, supporting the notion that PC1 represents tumour formation and that this is delayed in *Blg-Cre, Brca1*$^{f/f}$*;p53*$^{+/+}$ animals.

Supplementary Figure 3

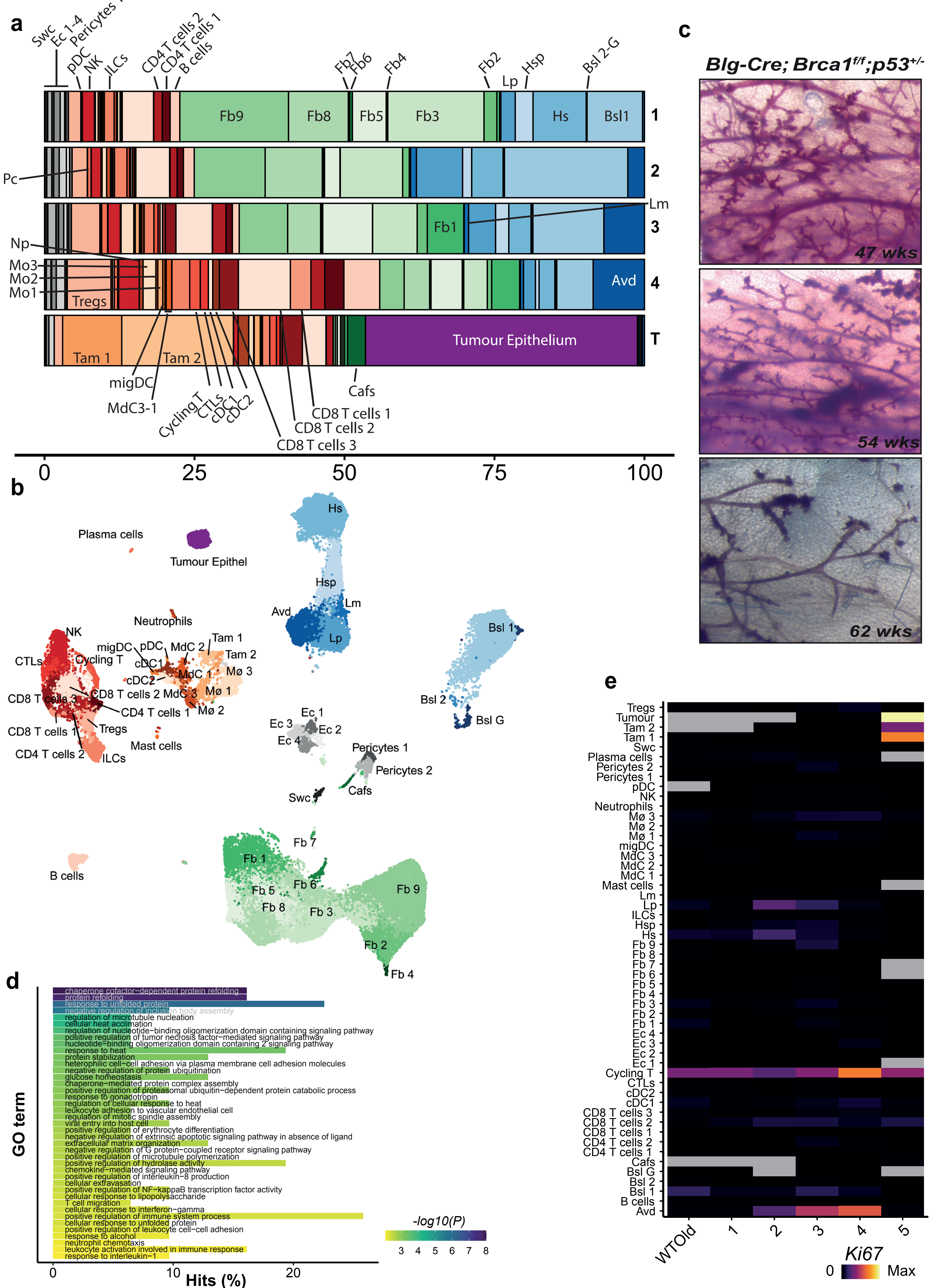

Supplementary Figure 3 Aberrant differentiation of luminal progenitors during tumourigenesis. (a) Same as in Figure 2B but with full annotation of all cell types. **(b)** UMAP as in Figure 1C with full cell type annotation. **(c)** Wholemounts of *Blg-Cre; Brca1*$^{f/f}$*; p53*$^{+/-}$ animals showing aberrant differentiation. **(d)** Gene set enrichment analysis of samples with high *CSN2* expression against all other samples. **(e)** Ki67 expression as an indicator of proliferative state in the various cell types found in the mouse samples.

Supplementary Figure 4

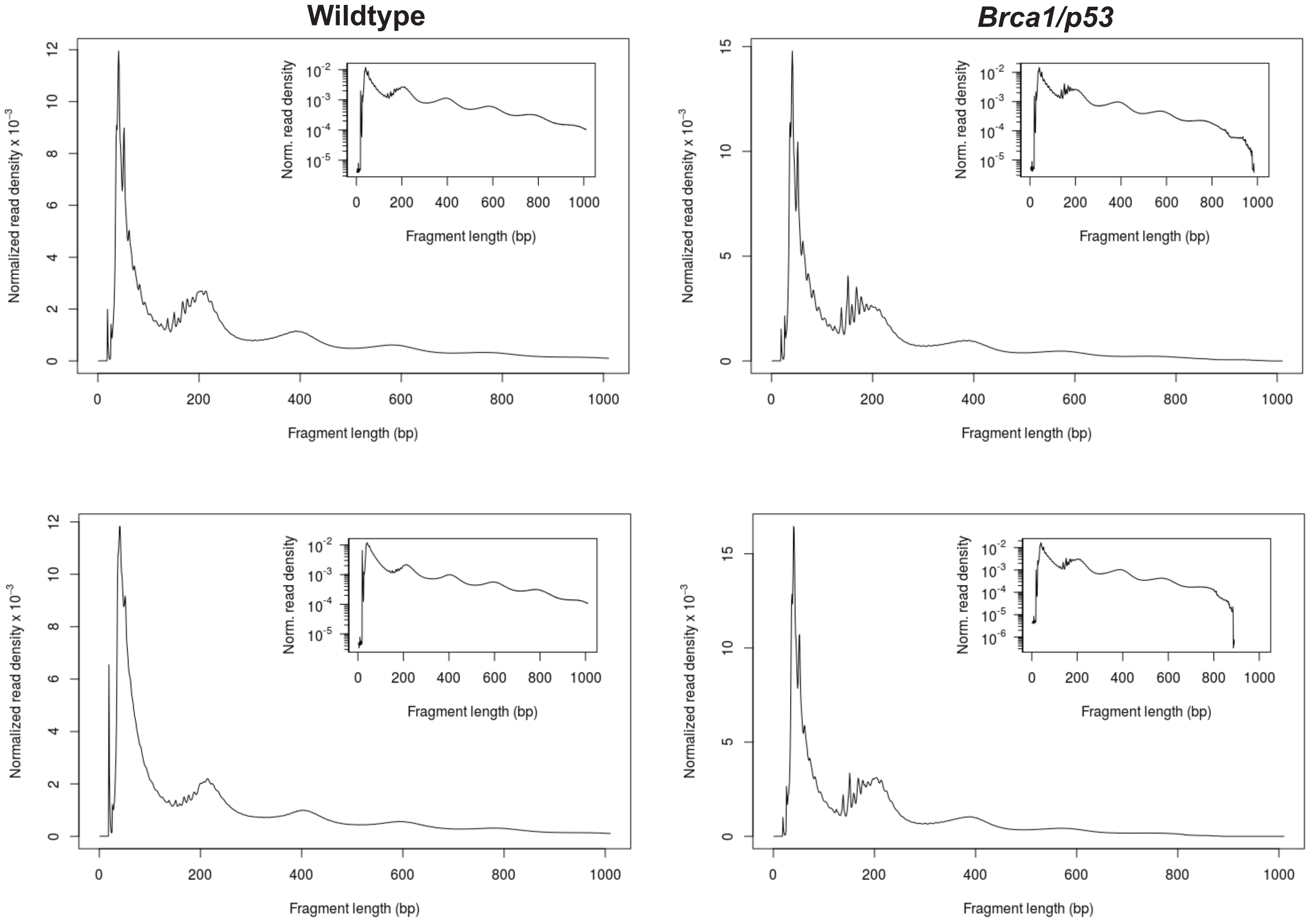

Supplementary Figure 4 Fragment size distribution of processed ATAC-Seq reads from wildtype and *Blg-Cre; Brca1$^{f/f}$; p53$^{+/-}$* samples. Enrichment of nucleosome-free (~100bp) and mono-nucleosome-bound fragments (~200bp) for the samples shown in Figure 2.

Supplementary Figure 5

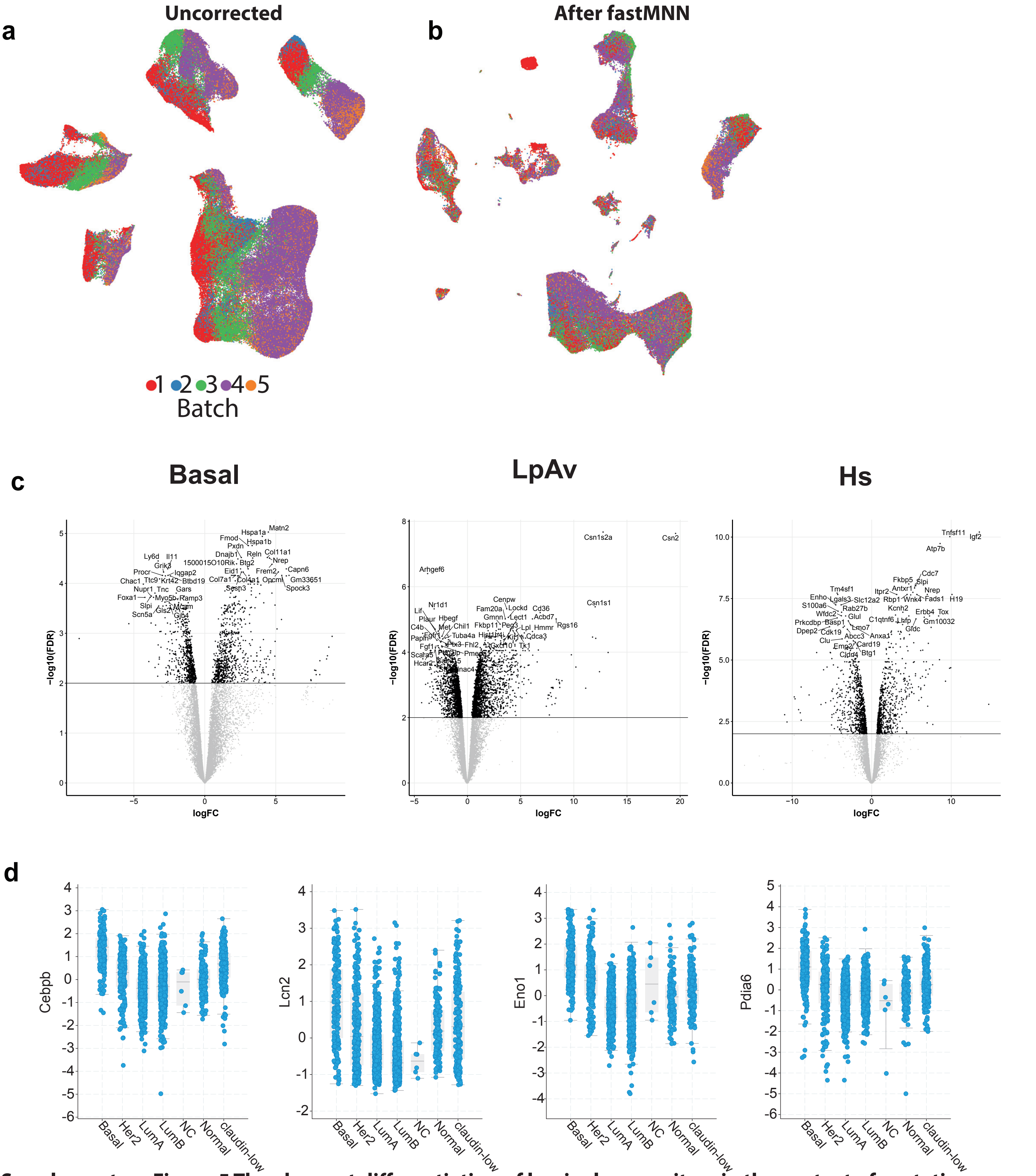

Supplementary Figure 5 The aberrant differentiation of luminal progenitors in the context of gestation. (**a,b**) UMAP before integration of the various batches (**a**) and after integrating the data with fastMNN (**b**). (**c**) Differential gene expression between gestation samples (14.5dG for Basal and LpAv, 9.5dG for Hs due to the lack of Hs cells at 14.5dG) and nulliparous samples. Positive logFC indiciates up-regulation during gestation. (**d**) mRNA expression z-Scores relative to all samples from the METABRIC study. X-axis depicts grouping into PAM50 + claudin low subtypes of 2509 cancer samples. The central line in the boxplot represents the median, the lower and upper hinges correspond to the first quartiles and the whisker extends from the hinge to the smalles/largest value no further than 1.5 x IQR from the hinge.

Supplementary Figure 6

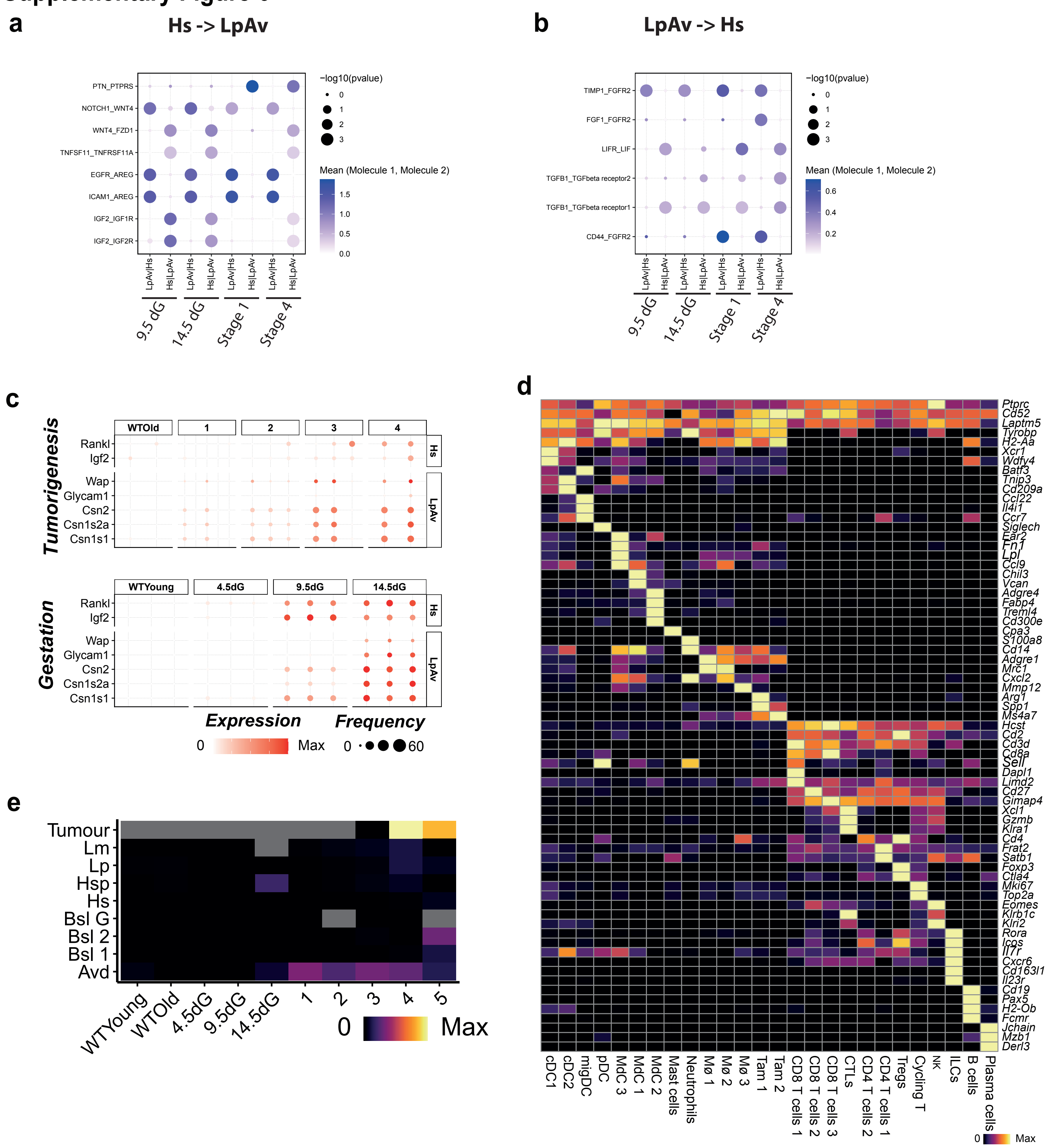

Supplementary Figure 6 Aberrant differentiation of luminal progenitor cells is accompanied by an altered micro-environment with tumour promoting characteristics. (**a**,**b**) CellphoneDB results for selected interactions from Hs cells to LpAv (**a**) and LpAv to Hs (**b**). The size of the dots corresponds to the FDR corrected P-Value and the colour to the mean expression of ligand and receptor. (**c**) Same data as presented in Figure 4C displayed as mean expression and frequency of expression per sample. (**d**) Marker gene expression for immune cells. (**e**) Gene expression of Spp1 as in Figure 4 including the tumour samples, expression scaled to 0-1.

Supplementary Figure 7

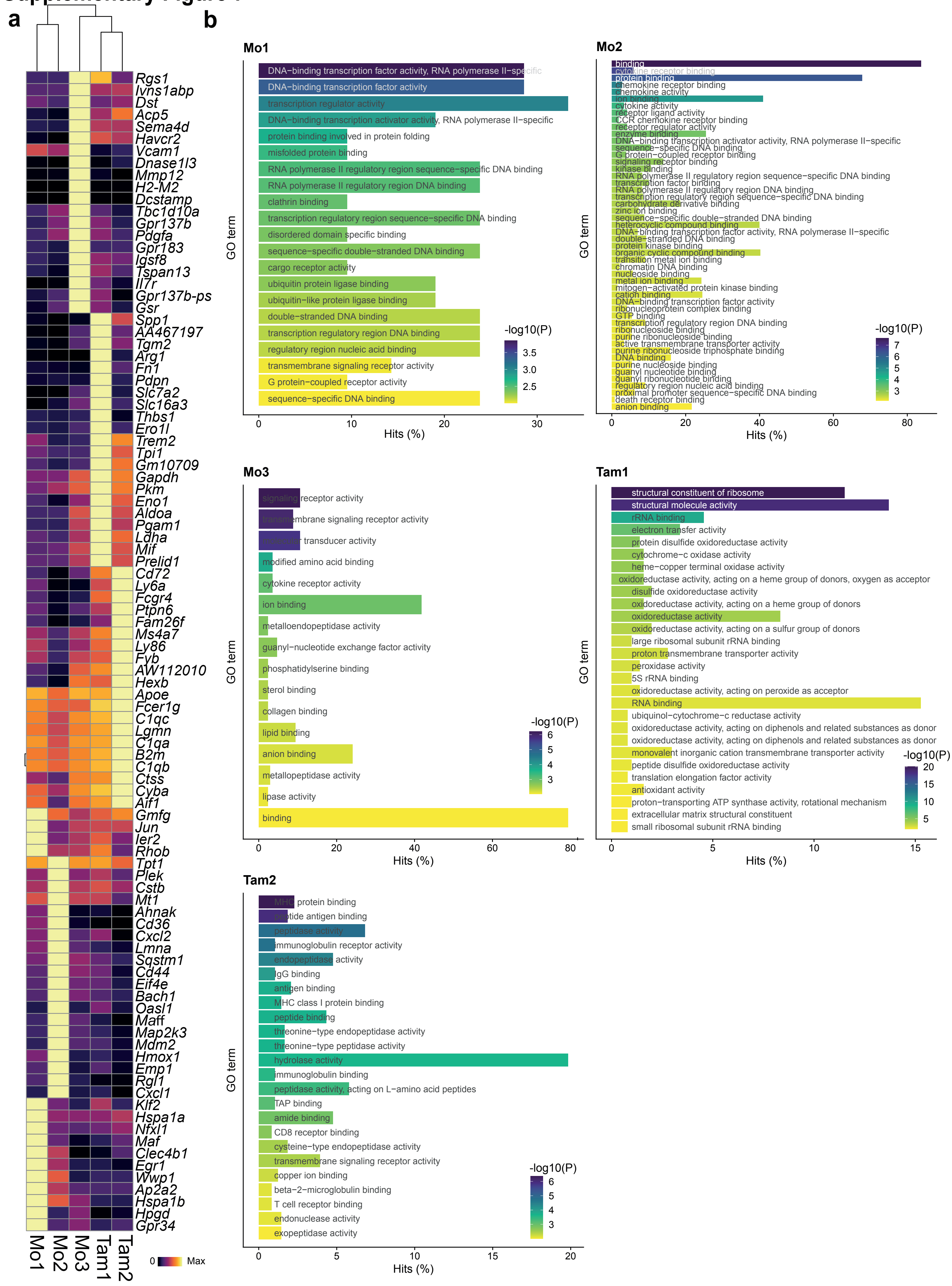

Supplementary Figure 7. Gene expression of the five macrophage populations. (a) Top genes differentially expressed between the five populations across all samples. **(b)** Molecular Function GO-Terms enriched in genes up-regulated against all other macrophages for each cluster at FDR <0.1.

Supplementary Figure 8

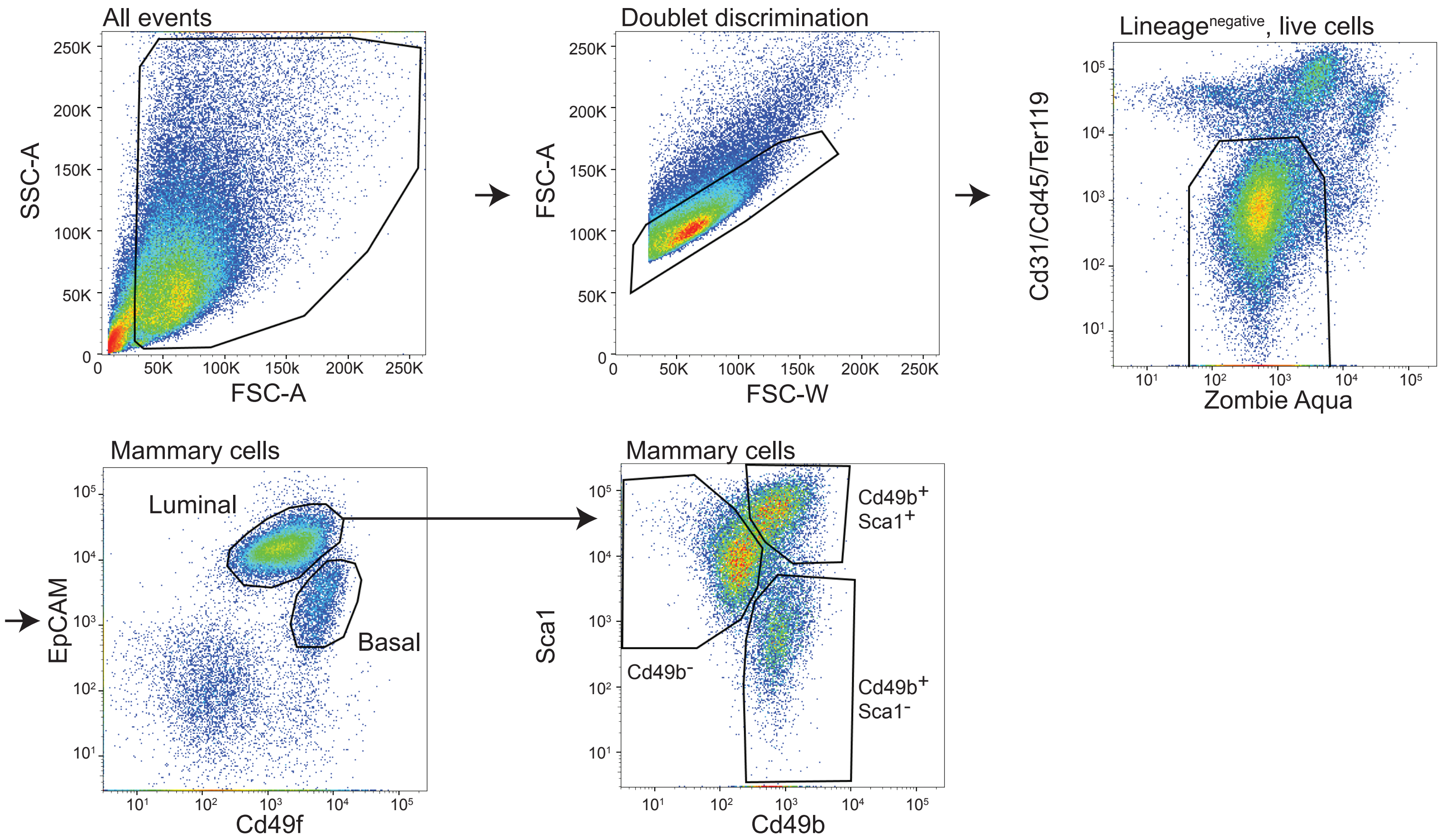

Supplementary Figure 8. Gating strategy used for mouse samples on the FACS Aria Fusion sorter. Representative plots showing the gating strategy used to select live, lineage negative, single cells in the mouse luminal and basal mammary epithelium, based on EpCAM and Cd49f staining of single cell preparations from lymph node divested mammary glands. FSC-W: forward scatter width, FSC-A: forward scatter area, SSC-A: side scatter area. Luminal differentiated and progenitor cells were identified based on Cd49b and Sca1 staining (Cd49b-, differentiated; Cd49b+ Sca1+ and Cd49b+ Sca1-, luminal progenitors). The arrows indicate sequential gating. This gating strategy was used in Figure 2g.

Supplementary Figure 9

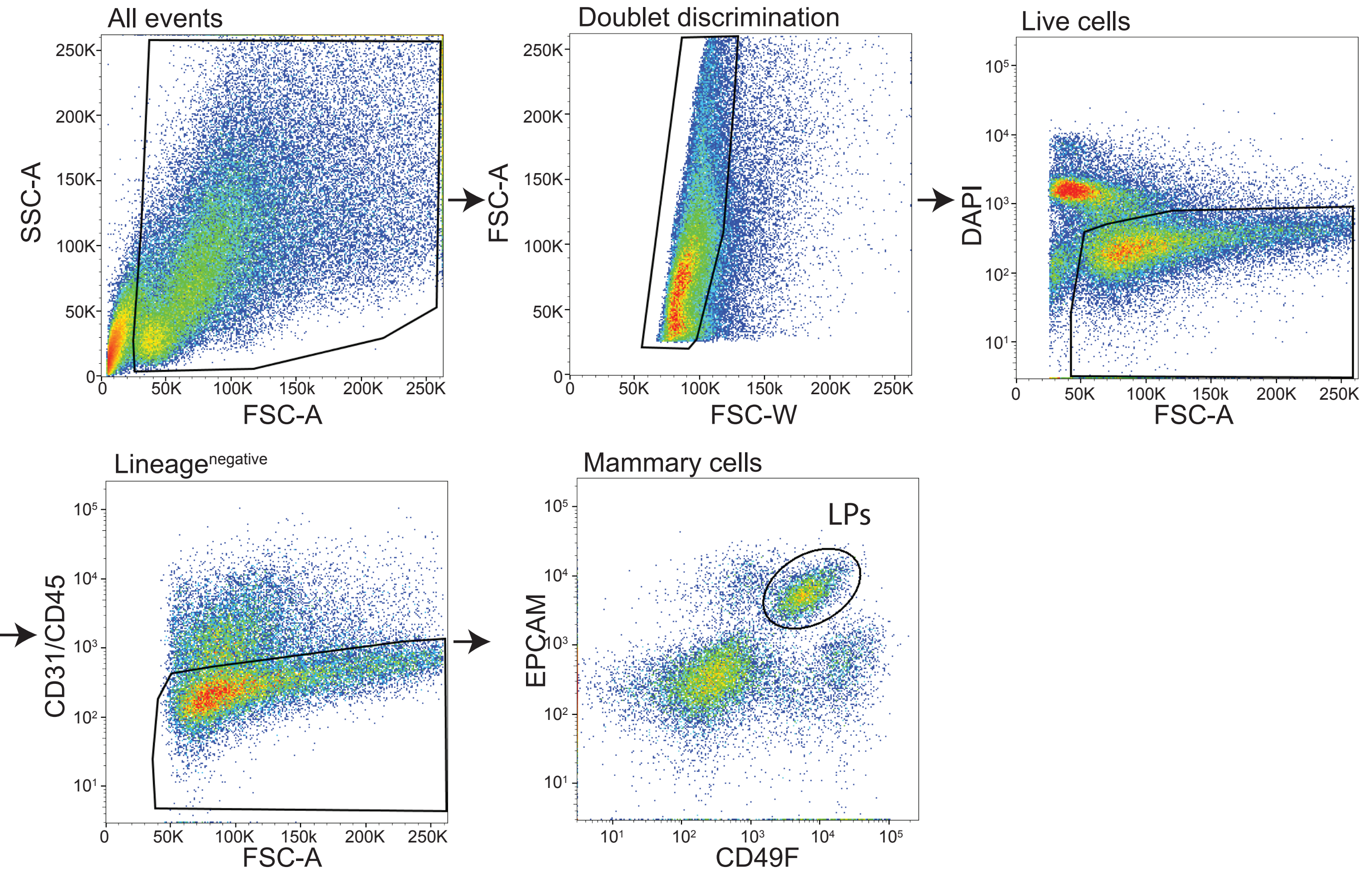

Supplementary Figure 9. Gating strategy used for human samples on the FACS Aria Fusion sorter. Representative plots showing the gating strategy used to select live, lineage negative, single luminal progenitor cells based on EPCAM and CD49F staining of single cell preparations.
FSC-W: forward scatter width, FSC-A: forward scatter area, SSC-A: side scatter area.
The arrows indicate sequential gating. This gating strategy was used in Figure 2h.

Supplementary Figure 10

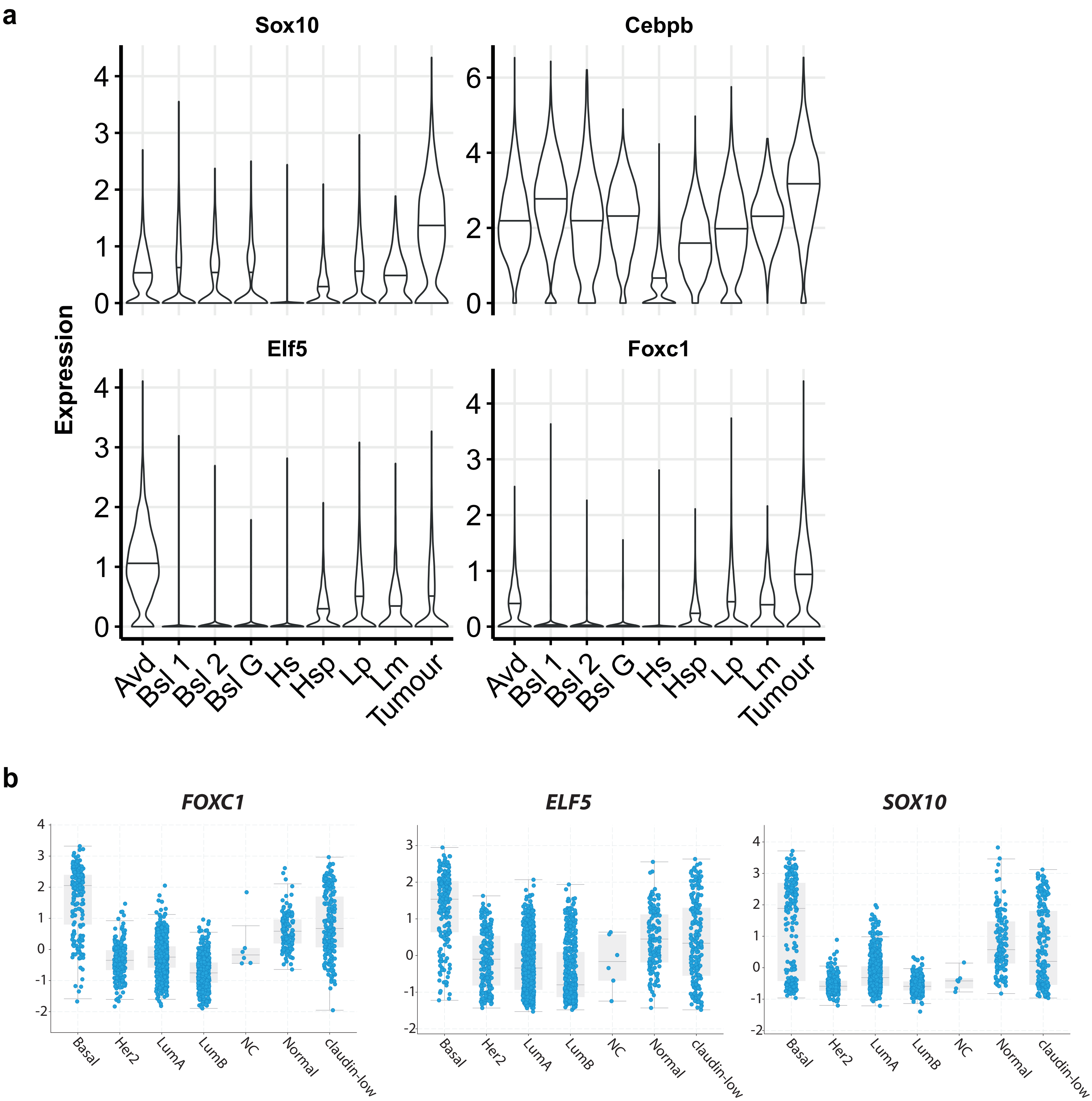

Supplementary Figure 10. Expression of transcriptional regulators of alveologenesis in tumours.
(**a**) Expression of selected transcription factors across the various epithelial compartments.
(**b**) mRNA expression z-Scores relative to all samples from the METABRIC study. X-axis depicts grouping into PAM50 + claudin low subtypes of 2509 cancer samples. The central line in the boxplot represents the median, the lower and upper hinges correspond to the first quartiles and the whisker extends from the hinge to the smalles/largest value no further than 1.5 x IQR from the hinge.
