# Supplementary material for: Time-resolved single-cell analysis of Brca1 associated mammary tumourigenesis reveals aberrant differentiation of luminal progenitors
Source: Nat Commun. 2021 Mar 9;12:1502. doi: 10.1038/s41467-021-21783-3 (PMC7940427; doi:10.1038/s41467-021-21783-3)
Supplement: Supplementary file 2 — Description of Additional Supplementary Files [file 41467_2021_21783_MOESM2_ESM.pdf]

Description of Additional Supplementary Files

Title: Supplementary Data 1

Description: Transcription factor binding motifs that are enriched in chromatin regions with higher accessibility in Brca1/p53 animals compared to WT.

Title: Supplementary Data 2

Description: Sequences of primers that were used for the genotyping of Brca1/p53 animals and probes that were used for qRT-PCR. mut=mutated locus, FW=forward, RV=reverse.

Title: Supplementary Data 3

Description: Clinical meta data of human samples.
