# Supplementary material for: Time-resolved single-cell analysis of Brca1 associated mammary tumourigenesis reveals aberrant differentiation of luminal progenitors
Source: Nat Commun. 2021 Mar 9;12:1502. doi: 10.1038/s41467-021-21783-3 (PMC7940427; doi:10.1038/s41467-021-21783-3)
Supplement: Supplementary file 6 — Reporting Summary [file 41467_2021_21783_MOESM6_ESM.pdf]

nature research

Corresponding author(s): Walid T Khaled, John C. Marioni

Last updated by author(s): Feb 4, 2021

Reporting Summary

Nature Research wishes to improve the reproducibility of the work that we publish. This form provides structure for consistency and transparency in reporting. For further information on Nature Research policies, see our Editorial Policies and the Editorial Policy Checklist.

Statistics

For all statistical analyses, confirm that the following items are present in the figure legend, table legend, main text, or Methods section.

n/a	Confirmed	
☐	☒	The exact sample size (n) for each experimental group/condition, given as a discrete number and unit of measurement
☐	☒	A statement on whether measurements were taken from distinct samples or whether the same sample was measured repeatedly
☐	☒	The statistical test(s) used AND whether they are one- or two-sided<br>*Only common tests should be described solely by name; describe more complex techniques in the Methods section.*
☐	☒	A description of all covariates tested
☐	☒	A description of any assumptions or corrections, such as tests of normality and adjustment for multiple comparisons
☐	☒	A full description of the statistical parameters including central tendency (e.g. means) or other basic estimates (e.g. regression coefficient) AND variation (e.g. standard deviation) or associated estimates of uncertainty (e.g. confidence intervals)
☐	☒	For null hypothesis testing, the test statistic (e.g. F, t, r) with confidence intervals, effect sizes, degrees of freedom and P value noted<br>*Give P values as exact values whenever suitable.*
☒	☐	For Bayesian analysis, information on the choice of priors and Markov chain Monte Carlo settings
☒	☐	For hierarchical and complex designs, identification of the appropriate level for tests and full reporting of outcomes
☒	☐	Estimates of effect sizes (e.g. Cohen's d, Pearson's r), indicating how they were calculated

Our web collection on statistics for biologists contains articles on many of the points above.

Software and code

Policy information about availability of computer code

Data collection	No software was used for data collection.
Data analysis	Cell Ranger Single-Cell Software Suite (3.10) was used for demultiplexing, barcode assignment and UMI quantification. All downstream computation analyses were performed in R (Version 3.4.1) using standard functions unless otherwise indicated.FlowJo V10 was used to analyse flow cytometry data.

For manuscripts utilizing custom algorithms or software that are central to the research but not yet described in published literature, software must be made available to editors and reviewers. We strongly encourage code deposition in a community repository (e.g. GitHub). See the Nature Research guidelines for submitting code & software for further information.

Data

Policy information about availability of data

All manuscripts must include a data availability statement. This statement should provide the following information, where applicable:

- Accession codes, unique identifiers, or web links for publicly available datasets
- A list of figures that have associated raw data
- A description of any restrictions on data availability

The authors declare that all data supporting the findings of this study and unprocessed images are available within the article and its supplementary information files or from the corresponding author upon reasonable request. The raw sequencing data is available on ArrayExpress with the following accession numbers: E-MTAB-10043 (scRNA-Seq) (https://www.ebi.ac.uk/arrayexpress/experiments/E-MTAB-10043/), E-MTAB-10046 (RNA-Seq) (https://www.ebi.ac.uk/arrayexpress/experiments/E-MTAB-10046/) and E-MTAB-10054 (ATAC-Seq) (https://www.ebi.ac.uk/arrayexpress/experiments/E-MTAB-10054/). Processed data can also be explored and downloaded at http://marionilab.cruk.cam.ac.uk/BRCA1Tumourigenesis.

Field-specific reporting

Please select the one below that is the best fit for your research. If you are not sure, read the appropriate sections before making your selection.

☒ Life sciences ☐ Behavioural & social sciences ☐ Ecological, evolutionary & environmental sciences

For a reference copy of the document with all sections, see nature.com/documents/nr-reporting-summary-flat.pdf

Life sciences study design

All studies must disclose on these points even when the disclosure is negative.

Sample size	No sample size calculation was performed.
Data exclusions	No data was excluded from the analyses
Replication	For the tumorigenesis scRNAseq data a total of 15 samples was collected, 13 pre-tumour samples, 2 WT controls, 2 tumours, each from individual mice. For the pregnancy scRNAseq dataset 12 samples were collected, 3 replicates per time-point. For th ATACseq data, 2 Brca1/p53 and 2 WT controls were collected, each from individual mice. For the human data, sample from 12 BRCA1 carriers and 12 reduction mammoplasties were used. For the representative immunofluorescence image shown in Figure 2F, 10 individual images from 3 independent replicates were analysed. Wholemounts from 8 individual Brca1/p53 mice were analysed and 4 representative images shown in Figure 2E and Supplementary figure 3C.
Randomization	No randomization was used as randomization is not applicable to the experimental setup.
Blinding	No blinding was used as investigators were aware of the genotypes and time points of all animals collected as well as the nature of the human samples.

Reporting for specific materials, systems and methods

We require information from authors about some types of materials, experimental systems and methods used in many studies. Here, indicate whether each material, system or method listed is relevant to your study. If you are not sure if a list item applies to your research, read the appropriate section before selecting a response.

Materials & experimental systems

n/a	Involved in the study	
☐	☒	Antibodies
☒	☐	Eukaryotic cell lines
☒	☐	Palaeontology and archaeology
☐	☒	Animals and other organisms
☐	☒	Human research participants
☒	☐	Clinical data
☒	☐	Dual use research of concern

Methods

n/a	Involved in the study	
☒	☐	ChIP-seq
☐	☒	Flow cytometry
☒	☐	MRI-based neuroimaging

Antibodies

Antibodies used	Mouse mammary cells were stained with the following primary antibodies: Cd31-biotin (eBioscience, clone 390, 1 µg ml−1, 1:500); Cd45-biotin (eBioscience, clone 30F11, 1 µg ml−1, 1:500); Ter119-biotin (eBioscience, clone Ter119, 1 µg ml−1, 1:500), EpCAM-APC/Cy7 (Biolegend, clone G8.8, 0.5 µg ml−1, 1:500), Cd49f-BV421 (Biolegend 313623, 2 µg ml−1, 1:100), Cd49b-AF488 (Biolegend, clone HMα2, 1 µg ml−1, 1:500) and Sca1-AF647 (Biolegend, clone D7, 1 µg ml−1, 1:500). Cells were then stained with Streptavidin-PE/Cy7 (BD-Biosciences, 0.4 µg ml−1, 1:500). Human mammary cells were stained with the following primary antibodies: CD45-APC (Biolegend, clone H130,1:100), CD31-APC (Biolegend, clone WM-59, 1:100), EPCAM-APC/Fire750 (Biolegend, clone 9C4, 1:50), CD49f-PE/Cy7 (Biolegend, clone GoH3, 1 µg ml−1, 1:200).
Validation	All antibodies used are commercially available.<br>Biolegend antibodies validation steps: https://www.biolegend.com/en-us/reproducibility<br>eBioscience (now Thermo Fisher Scientific) validation steps: https://www.thermofisher.com/uk/en/home/life-science/antibodies/invitrogen-antibody-validation.html<br>BD-Biosciences: https://www.biocompare.com/Antibody-Manufacturing/355107-Antibody-Manufacturing-Perspectives-BD-Bioscience/

Animals and other organisms

Policy information about studies involving animals; ARRIVE guidelines recommended for reporting animal research

Laboratory animals	The Blg-Cre;Brca1f/f;p53+/- (JAX 012620)19 mouse model was used to study TNBC tumour development. In detail, tissues were collected from 15 nulliparous mice with age ranging from 30 to 48 weeks (Supplementary Fig. 1A). At time of collection, 13 mice showed no presence of tumours, while 2 presented tumours in one of the glands. In addition, we collected glands from two Blg-Cre;Brca1f/f;p53+/+ that were used as validation for the ordering of the samples (Supplementary Fig. 2D). For the tumour-bearing mice, contralateral glands and tumours cleared of surrounding mammary gland tissue were treated as independent samples in the data set. For the pregnancy time points, females were mated with studs. Tissues were then harvested from 3 individual mice per time point at gestation day 4.5, 9.5 and 14.5. Tissue from nulliparous wildtype females was harvested at 12 weeks of age for comparison to the pregnancy time points (Young nulliparous, n=3), and at 53 and 74 weeks of age for comparison to the premalignant and tumour stages (Old nulliparous). For the ATAC-Seq experiment, 2 wildtype and 2 Blg-Cre;Brca1f/f;p53+/- mice (aged between 36 and 40 weeks) were used. All mice were housed in individually ventilated cages under a 12:12h light–dark cycle, with water and food available ad libitum
Wild animals	This study did not include wild animals
Field-collected samples	This study did not include field-collected samples
Ethics oversight	All experimental animal work was performed in accordance to the Animals (Scientific Procedures) Act 1986, UK and approved by the Ethics Committee at the Sanger Institute.

Note that full information on the approval of the study protocol must also be provided in the manuscript.

Human research participants

Policy information about studies involving human research participants

Population characteristics	All primary human breast tissue was derived from women undergoing reduction mammoplasties with no known genetic history (n = 12) and prophylactic mastectomies from women with germline BRCA1 mutations (n = 12, one of which had a tumour in the contralateral gland). No specific age-range was selected.
Recruitment	Participants were not specifically recruited for this study and are part of bigger cohorts where recruitment was not based on the parameters of interest for this analysis.
Ethics oversight	All primary human breast tissue was derived from women undergoing reduction mammoplasties with no known genetic history (n = 12) and prophylactic mastectomies from women with germline BRCA1 mutations (n = 12, one of which had a tumour in the contralateral gland) under full informed consent either at Addenbrooke's Hospital, Cambridge, UK, in accordance with the National Research Ethics Service, Cambridgeshire 2 Research Ethics Committee approval (08/H0308/178) as part of the Adult Breast Stem Cell Study or obtained from the Breast Cancer Now Tissue bank, as approved by Cambridge Central REC (15/EE/0192).

Note that full information on the approval of the study protocol must also be provided in the manuscript.

Flow Cytometry

Plots

Confirm that:

- [x] The axis labels state the marker and fluorochrome used (e.g. CD4-FITC).
- [x] The axis scales are clearly visible. Include numbers along axes only for bottom left plot of group (a 'group' is an analysis of identical markers).
- [x] All plots are contour plots with outliers or pseudocolor plots.
- [x] A numerical value for number of cells or percentage (with statistics) is provided.

Methodology

Sample preparation	Lymph node divested mouse mammary glands (excluding the cervical pair) were mechanically dissociated after collection, pooled per animal and the finely minced tissue was transferred to DMEM/F12 (Gibco)+10mM HEPES (Gibco)+2 mg ml-1 collagenase (Roche)+200 U ml−1 hyaluronidase (Sigma) (CH)+gentamicin (Gibco) at 37°C and vortexed every 30min. After the lysis of red blood cells in NH4Cl, cells were briefly digested with warm 0.05% Trypsin-EDTA (Gibco), 5mg ml−1 dispase (Sigma) and 1mg ml−1 DNase (Sigma) and filtered through a cell strainer (BD Biosciences).<br>Frozen vials of human epithelial-enriched fractions dissociated as previously described20 or of organoids from the Breast Cancer Now tissue bank were defrosted and diluted in cold HBSS 1% FCS (HF), further digested with warm Trypsin-EDTA (Gibco), 5mg ml−1 dispase (Sigma) and 1mg ml−1 DNase (Sigma) and filtered through a 40M cell strainer (BD Biosciences).
Instrument	FACS Aria Fusion

Software	FlowJo
Cell population abundance	Purity of samples was not determined post-sorting.
Gating strategy	After doublets, dead cells and contaminating haematopoietic, endothelial and stromal cells were sequentially manually gated out adapting gate positioning on each individual sample appearance, human luminal progenitors for RNA processing and mouse CD49b+, Sca1- luminal progenitors for ATAC-Seq were sorted. The gating strategies are reported in Supplementary Fig. 8 and 9. Starting cell population values for FSC include all cells between 25k and 250k, and the whole range of SSC was included.

☒ Tick this box to confirm that a figure exemplifying the gating strategy is provided in the Supplementary Information.
